# Mapping of host-parasite-microbiome interactions reveals metabolic determinants of tropism and tolerance in Chagas disease

**DOI:** 10.1126/sciadv.aaz2015

**Published:** 2020-07-22

**Authors:** E. Hossain, S. Khanam, D. A. Dean, C. Wu, S. Lostracco-Johnson, D. Thomas, S. S. Kane, A. R. Parab, K. Flores, M. Katemauswa, C. Gosmanov, S. E. Hayes, Y. Zhang, D. Li, C. Woelfel-Monsivais, K. Sankaranarayanan, L.-I. McCall

**Affiliations:** 1 Department of Chemistry and Biochemistry, University of Oklahoma, Norman, OK 73019, USA.; 2 Laboratories of Molecular Anthropology and Microbiome Research, University of Oklahoma, Norman, OK 73019, USA.; 3 Department of Microbiology and Plant Biology, University of Oklahoma, Norman, OK 73019, USA.; 4 Skaggs School of Pharmacy and Pharmaceutical Sciences, University of California San Diego, La Jolla, CA 92093, USA.; 5 Department of Biology, University of Oklahoma, Norman, OK 73019, USA.; 6 College of Chemistry, Beijing Normal University, Beijing 100875, China.

## Abstract

Chagas disease (CD) is a parasitic disease caused by *Trypanosoma cruzi* protozoa, presenting with cardiomyopathy, megaesophagus, and/or megacolon. To determine the mechanisms of gastrointestinal (GI) CD tissue tropism, we systematically characterized the spatial localization of infection-induced metabolic and microbiome alterations, in a mouse model of CD. Notably, the impact of the transition between acute and persistent infection differed between tissue sites, with sustained large-scale effects of infection in the esophagus and large intestine, providing a potential mechanism for the tropism of CD within the GI tract. Infection affected acylcarnitine metabolism; carnitine supplementation prevented acute-stage CD mortality without affecting parasite burden by mitigating infection-induced metabolic disturbances and reducing cardiac strain. Overall, results identified a previously-unknown mechanism of disease tolerance in CD, with potential for new therapeutic regimen development. More broadly, results highlight the potential of spatially resolved metabolomics to provide insight into disease pathogenesis and infectious disease drug development.

## Teaser

Chemical cartography of experimental gastrointestinal Chagas disease identifies previously-unknown mechanisms of disease tolerance.

## INTRODUCTION

Chagas disease (CD), also known as American trypanosomiasis, is a neglected tropical disease endemic in Latin America. However, because of migration, CD now has a global reach spanning North America, Europe, and Asia. CD is caused by infection with the protozoan parasite *Trypanosoma cruzi*. Six to 8 million people are infected with *T. cruzi*, with approximately 12,000 deaths per year. Infected individuals pass first through an acute disease stage, usually asymptomatic, and then to a chronic asymptomatic (indeterminate) stage that can last for decades. Thirty to 40% of infected individuals progress from indeterminate to determinate (symptomatic) chronic CD, 20 to 30% with cardiovascular complications (heart failure, arrhythmias, and thromboembolism) and 10 to 15% with gastrointestinal (GI) symptoms (megaesophagus and megacolon) (*1*). Digestive CD has been neglected compared to cardiac CD and is consequently much more poorly understood. However, recent studies using bioluminescent parasites in mouse models have shown that specific sites in the GI tract are parasite reservoirs in chronic CD and may be major contributors to cardiac symptom development, particularly after treatment failure (*2*, *3*). Treatment of GI CD is also challenging, with limited options once symptoms become apparent (*1*). There is, therefore, a strong need to improve our understanding of the interaction between *T. cruzi* and the GI tract, both to clarify mechanisms of GI CD pathogenesis and to define GI factors contributing to cardiac CD, leading to new treatment strategies.

The GI tract is a complex environment where host, pathogen, and microbiota interact to affect disease pathogenesis. We previously demonstrated that *T. cruzi* infection affects the fecal microbiome and metabolome (*4*), but information on the specific GI sites driving this output had not yet been determined. In this study, we integrated small molecule–focused liquid chromatography–tandem mass spectrometry (LC-MS/MS) and spatial metabolite mapping (“chemical cartography”), in conjunction with microbiome analysis, to systematically characterize the *T. cruzi*–induced changes in the GI microenvironment in acute and persistent, long-term, experimental *T. cruzi* infection. We specifically focused on small-molecule characterization because small molecules represent the output of cellular processes as well as their regulators and therefore have the closest relationship to phenotype (*5*). Given that most drugs are still small molecule–based, we further hypothesized that identifying infection-associated disturbances in the small-molecule profile can most rapidly lead to new treatments for CD.

Results identified organ-specific and organ subsite–specific disruptions in the chemical and microbial GI environment by *T. cruzi* and highlighted differential mechanisms of transition from acute to persistent, long-term infection depending on the organ. These results provide a mechanism by which consistent perturbations of tissue biochemical pathways lead to GI CD pathology in the esophagus and large intestine. Consistent infection-induced elevation of acylcarnitine family members across organs further led us to investigate the role of acylcarnitines in disease pathogenesis. Supplementing animal drinking water with carnitine prevented acute-stage mortality in experimental CD in the absence of antiparasitic effect, revealing a previously-unknown mechanism of disease tolerance in CD, via resetting of host metabolism back toward a metabolic profile similar to uninfected animals. Overall, these results identified previously-unknown mechanisms of CD pathogenesis, with major translational applications to CD drug development. Furthermore, the data collected here on uninfected animals, and our approach in general, can serve as a reference to investigate determinants of pathogen tropism and novel treatment strategies for any other GI pathogen.

## RESULTS

### Regiospecific molecular impact of *T. cruzi* colonization in the GI tract

**Fig. 1:**
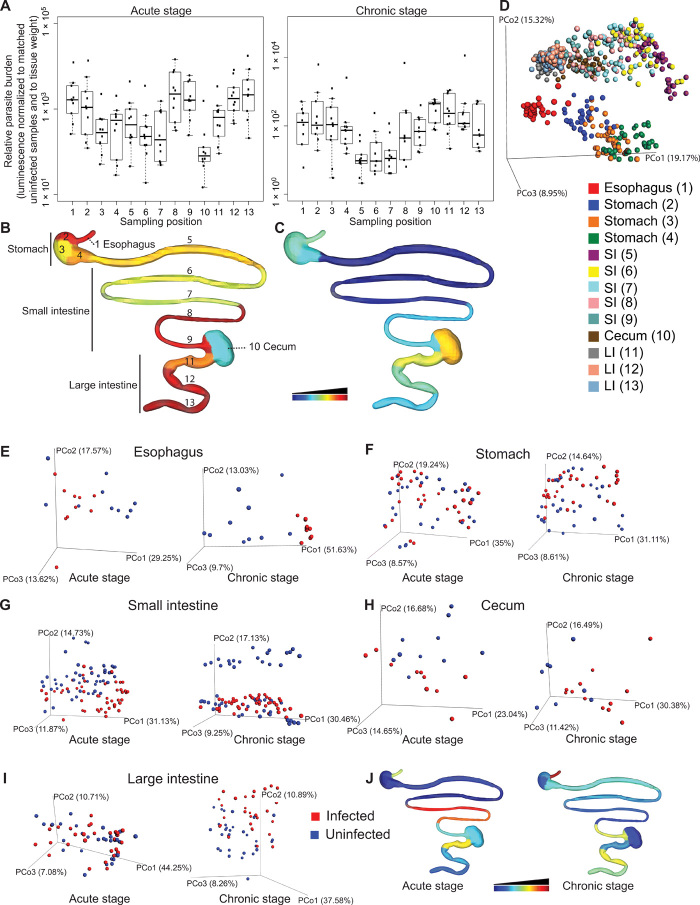
Spatial impact of *T. cruzi* infection is reflected by spatial modulation of the tissue small-molecule profile. C3H/HeJ male mice (*n* = 5 per group and replicate) were mock-infected or infected with 1000 luminescent *T. cruzi* strain CL Brener trypomastigotes in two biological replicates. GI samples were collected 12 and 89 days after infection. (**A**) Parasite burden at each sampling site. To correct for variations in sample size and background signal, luminescence counts were normalized to signal from matched uninfected samples and to sample weight, for each sampling position. (**B** and **C**) Median normalized luminescent signal, at each sampling site, 12 days after infection (B) and 89 days after infection (C). Common logarithmic scale for (B) and (C), scaled from lowest luminescent signal (dark blue) to highest signal (dark red). (**D**) PCoA analysis showing separation between sampling sites in terms of overall chemical composition, even within a given organ (positive mode, all time points combined, Bray-Curtis-Faith distance metric). (**E** to **I**) PCoA analysis showing chemical composition differences (positive mode analysis) between infected and uninfected samples in the esophagus (E), stomach (F), small intestine (G), cecum (H), and large intestine (I). (**J**) *R*^2^ at each sampling site [common logarithmic scale, scaled from lowest *R*^2^ (dark blue) to highest *R*^2^ (dark red)]. PCo, principal coordinate (e.g., PCo1, principal coordinate 1).

GI CD is still poorly understood. In our prior work, we identified specific small molecules correlated with cardiac parasite tropism (*6*). Here, we sought to identify the local tissue chemical changes associated with parasite GI colonization. Mice were infected with 1000 luciferase-expressing *T. cruzi* strain CL Brener parasites [strain generated and provided by J. Kelly, London School of Hygiene & Tropical Medicine (*3*)]. Twelve days (acute stage) and 89 days after infection (persistent, long-term infection), animals were euthanized, the GI tract was sectioned, and parasite burden in each section was determined by ex vivo bioluminescence imaging (Fig. 1, A to C, and fig. S1). At 12 days after infection, parasite burden was high throughout the GI tract, except in the cecum, where the parasite burden was significantly lower than in the esophagus (Kruskal-Wallis *P* < 0.05 for impact of sampling position on parasite burden and Dunn’s post hoc test with Bonferroni correction, *P* = 0.0075), in the distal small intestine (positions 8 and 9, Dunn’s post hoc test with Bonferroni correction, *P* = 0.0037 and *P* = 0.0052, respectively), and in the distal large intestine (positions 12 and 13, Dunn’s post hoc test with Bonferroni correction, *P* = 0.0032 and *P* = 0.0014, respectively; Fig. 1, A and B). At 89 days after infection, the parasite burden was significantly higher in the cecum and in parts of the large intestine than in the small intestine (Kruskal-Wallis *P* < 0.05 for impact of sampling position on parasite burden and Dunn’s post hoc test with Bonferroni correction, *P* = 0.00037, *P* = 0.0034, and *P* = 0.0086, for positions 10, 11, and 12 compared to position 5, respectively; *P* = 0.0055 and *P* = 0.036 for positions 10 and 11 compared to position 6, respectively; and *P* = 0.0016, *P* = 0.013, and *P* = 0.030 for positions 10, 11, and 12 compared to position 7, respectively; Fig. 1, A and C). In general, as expected, parasite burden decreased from 12 to 89 days after infection. However, unexpectedly, parasite burden increased in the cecum during the transition from acute to persistent disease, suggesting a possible role for the cecum as a parasite reservoir protected from antiparasitic immune responses (Fig. 1, A to C). These observations indicate that all GI sites can initially harbor *T. cruzi* but then differentially respond to parasite presence, leading to the ability of the parasite to persist in some sites but not others.

Given this differential parasite tropism in persistent infection and the unique aspects of CD pathology, we sought to investigate the molecular determinants of parasite persistence versus disease resolution. To do so, we extracted small molecules (metabolites) from each collected GI section and analyzed these molecules by LC-MS/MS in positive and in negative mode. As expected, the strongest determinant of overall chemical profile was the source organ and sample position within that organ, as observed by principal coordinate analysis (PCoA; Fig. 1D). In PCoA analysis, each sample is represented as a sphere in three-dimensional (3D) space, with spheres close together indicating samples with similar metabolite composition. Statistical significance of PCoA clustering was evaluated using permutational multivariate analysis of variance (PERMANOVA), with PERMANOVA *P* value <0.05 indicating statistical significance and PERMANOVA *R*^2^ indicating the percentage of chemical variation that is explained by specified metadata variables. Sampling position and source organ thus had a significant impact on the tissue metabolite composition [PERMANOVA based on sampling position, *P* < 0.001, *R*^2^ = 12.067% (all time points combined); PERMANOVA based on source organ, *P* < 0.001, *R*^2^ = 39.951% (all time points combined)]. In contrast, PERMANOVA analyses based on infection status had lower *R*^2^ values, indicating the absence of organism-wide shifts in metabolite composition in response to infection (12 days after infection, *P* = 0.019, *R*^2^ = 1.011% and 89 days after infection, *P* = 0.014, *R*^2^ = 0.982%; both time points combined, *P* = 0.008, *R*^2^ = 0.598%). Comparison of chemical families differentially modulated by infection also identified few commonalities between sample sites (fig. S2). We therefore focused our analysis on the impact of infection in each individual organ.

Visualization of the chemical profile in each organ in relationship to infection status using PCoA analysis revealed organ-specific differences in the impact of *T. cruzi* infection. Acute-stage infection was associated with major disturbances in the overall esophagus chemical profile (PERMANOVA *P* = 0.002, *R*^2^ = 15.871%), with lower-scale perturbations in the small intestine and cecum (PERMANOVA *P* < 0.001, *R*^2^ = 6.95% and PERMANOVA *P* = 0.02, *R*^2^ = 10.411%, respectively) (Fig. 1E). The strongest acute-stage disruption within the small intestine chemical environment was observed in the distal small intestine, where parasite burden is the highest (PERMANOVA *P* < 0.001, *R*^2^ = 30.198%; *P* < 0.001, *R*^2^ = 26.063; and *P* = 0.006, *R*^2^ = 12.564, for sampling positions 7, 8, and 9, respectively; Fig. 1J). Metabolic disruptions that had been observed in the acute stage resolved by 89 days after infection for the cecum (PERMANOVA *P* = 0.17, *R*^2^ = 6.955%), decreased in magnitude for the small intestine (PERMANOVA *P* < 0.001, *R*^2^ = 4.824), became apparent in the stomach and large intestine (PERMANOVA *P* = 0.021, *R*^2^ = 4.429% and PERMANOVA *P* = 0.008, *R*^2^ = 6.323%, respectively), and increased in magnitude in the esophagus (PERMANOVA *P* < 0.001, *R*^2^ = 38.061%; Fig. 1, E to I). The largest statistically significant sites of metabolome disturbance in later disease stages were the esophagus and large intestine, which are the sites of damage in symptomatic human chronic-stage GI CD (*1*). On a per-sampling site basis, spatial heterogeneity in terms of overall effect size (*R*^2^) was observed within a given organ. The largest increase in *R*^2^ during the transition from acute to persistent infection was observed in the esophagus, distal stomach, and central large intestine (2.4-, 2.0-, and 1.9-fold increases, respectively; Fig. 1J).

**Fig. 2:**
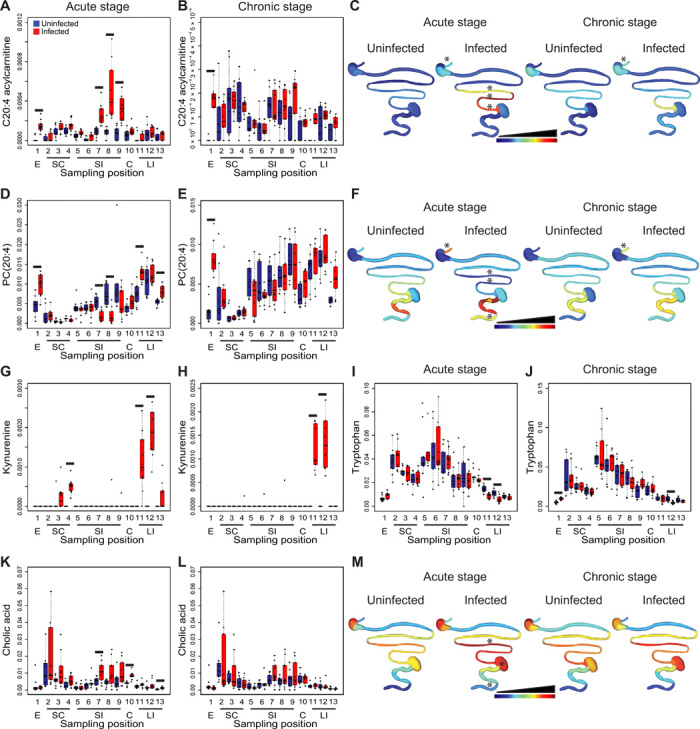
Common and tissue-specific metabolic changes identified by random forest demonstrate persistence of these alterations at sites of CD. (**A** to **C**) Infection-induced elevation of C20:4 acylcarnitine in the esophagus and small intestine in the acute stage, persisting in the esophagus in the later infection stage. (**D** to **F**) Infection-induced elevation of PC(20:4) in the esophagus and large intestine in the acute stage, persisting in the esophagus 89 days after infection. PC(20:4) was decreased in the acute stage in the infected small intestine. (**G** and **H**) Infection-induced elevation of kynurenine in stomach and large intestine in the acute stage, persisting in the large intestine 89 days after infection. (**I** and **J**) Infection-induced decrease in tryptophan at sites of increased kynurenine in the large intestine (12 and 89 days after infection). Infection also increased tryptophan in the esophagus 89 days after infection. (**K** to **M**) Infection-induced increase in the small intestine, cecum, and large intestine cholic acid (all detected adducts combined), acute stage only. E, esophagus; SC, stomach; SI, small intestine; C, cecum; LI, large intestine. Black lines in (A and B), (D and E), and (G to L) indicate false discovery rate (FDR)–corrected Mann-Whitney *P* < 0.05. Spatial distribution plots in (C), (F), and (M) are scaled for each specified metabolite, from lowest median normalized metabolite abundance (dark blue) to highest abundance (dark red). Linear scale in (C) and (F) and logarithmic scale in (M). Asterisks (*) above GI tract positions in (C), (F), and (M) indicate sites with FDR-corrected Mann-Whitney *P* < 0.05 compared to matched uninfected samples.

Next, we investigated the nature of the specific metabolic changes associated with these infection-altered overall chemical profiles. Feature annotation rates were considerably higher in positive mode than in negative mode (35.4 versus 10.2%), so we focused this analysis on positive mode LC-MS/MS data. We used machine learning (random forest) approaches to identify specific molecular features driving the differences between infected and uninfected tissues. Given our observations on the impact of sampling position on metabolite features (PCoA; Fig. 1D), these comparisons were independently performed for each organ. In the acute stage, we observed elevation in specific acylcarnitines and specific phosphatidylcholine (PC) family members in the different organs (Fig. 2, A to F; data file S1; and fig. S3). We also observed elevation in kynurenine in the stomach and large intestine in the acute stage. These differences continued at 89 days after infection for the proximal and central large intestine only (Fig. 2, G and H; data file S1; and fig. S3). Notably, the levels of tryptophan, the precursor of kynurenine, were correspondingly decreased in the acute stage at the same large intestine sites where kynurenine was elevated, whereas it was increased by infection in persistent infection in the esophagus (Fig. 2, I and J). Kynurenine is induced by inflammation; kynurenine metabolites have direct antiparasitic effects and contribute to the control of acute *T. cruzi* infection (*7*). However, they can also induce regulatory T cells (*7*), and hence, our observation of continued kynurenine elevation at later time points in the large intestine may contribute to parasite persistence in this organ. In accordance with our prior observations in the context of the fecal metabolome (*4*), specific large intestine and small intestine bile acid derivatives were increased in infected mice at 12 days after infection (Fig. 2, K and M; data file S1; and fig. S3). At 89 days after infection, molecular features identified as elevated by infection include specific acylcarnitines (e.g., C20:4 acylcarnitine in the esophagus), specific PCs [e.g., PC(22:5), PC(20:4), and PC(22:6) in the esophagus and PC(22:4) in the large intestine], specific amino acids, and derivatives (e.g., kynurenine in the large intestine and tryptophan in the esophagus) (Fig. 2, B to J; data file S1; and fig. S3). The pattern of resolution versus persistence of these metabolic changes reflected known sites of CD: For example, most of the top 10 metabolic perturbations observed in the acute stage in the esophagus were still perturbed 89 days after infection, whereas all the small intestine acute-stage perturbations were resolved in later disease stages (Fig. 2 and data file S1). Lysophosphatidylcholines (LPCs), in particular, increase *T. cruzi* invasion and inflammatory cell recruitment while inhibiting antiparasitic nitric oxide production (*8*). Thus, the sustained PC elevation observed in the esophagus and large intestine may contribute to CD progression in those organs. Overall, these results identified tissue metabolic changes linked to GI infection distribution and to sites of CD in the GI tract, as well as several metabolic pathways correlated with infection status.

### Durable effect of *T. cruzi* colonization on the GI tract microbiome

Several of the molecules identified in our dataset are of microbial origin or are microbially modified, such as indole-l-lactate, indoxyl sulfate, and secondary bile acids. Studies comparing germ-free and colonized mice have also shown that the microbiota influences a variety of the metabolites detected in our study, including bile acids, tryptophan, tyrosine, and maltotriose (*9*, *10*). Tryptophan and tyrosine, in particular, were affected by infection [tryptophan: decreased in the large intestine overall, Mann-Whitney *P* = 6.8 × 10^−6^ (12 days after infection) and *P* = 0.0043 (89 days after infection); tyrosine: decreased in the large intestine overall, Mann-Whitney *P* = 0.015 (acute stage), nonsignificant (89 days after infection)]. We have previously demonstrated that experimental *T. cruzi* infection alters the fecal microbiome and metabolome (*4*), a finding that was recently confirmed in *T. cruzi–*infected children in Bolivia (*11*). We therefore sought to evaluate the spatial impact of *T. cruzi* infection on the microbiota at each collection site in acute-stage disease (except for the esophagus where insufficient material was available to perform both metabolomic and 16*S* analyses) and focusing on the cecum and large intestine in persistent infection, given their role as major sites of CD pathogenesis and the unique metabolomic pattern observed at these sites (Fig. 1, H to J).

**Fig. 3:**
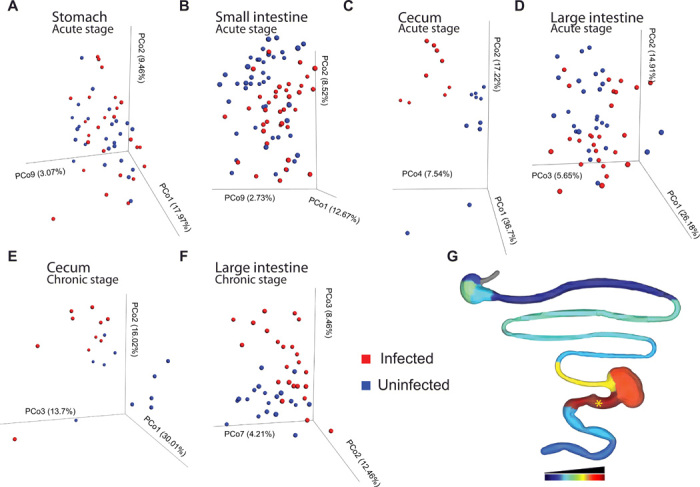
*T. cruzi* infection has a persistent, spatially heterogeneous impact on the microbiota. Representation of between-sample differences in microbial community composition through principal coordinate transformation of unweighted UniFrac distances. (**A** to **D**) Comparison of acute-stage infected and uninfected samples from stomach (A), small intestine (B), cecum (C), and large intestine (D). (**E** and **F**) Comparison of cecum (E) and large intestine (F) samples from uninfected and persistently infected mice. Spatial heterogeneity was also observed within an organ (G), with the highest disturbances in the microbiota in the proximal large intestine (sampling position 11). (**G**) *R*^2^ at each sampling site in the acute stage [logarithmic scale, scaled from lowest *R*^2^ (dark blue) to highest *R*^2^ (dark red)]. *PERMANOVA *P* < 0.05.

Differences in the overall microbiota composition (beta diversity, all sites combined for a given organ) were observed in the stomach and large intestine in the acute stage (PERMANOVA *P* = 0.05, *R*^2^ = 3.22% and PERMANOVA *P* = 0.04, *R*^2^ = 3.837%, respectively), with nonsignificant changes in the small intestine and cecum (PERMANOVA *P* = 0.069, *R*^2^ = 1.791% and PERMANOVA *P* = 0.058, *R*^2^ = 10.657%, respectively). These differences increased in magnitude for the cecum and large intestine during the transition from acute to persistent infection (PERMANOVA *P* = 0.02, *R*^2^ = 11.556% and PERMANOVA *P* = 0.002, *R*^2^ = 5.83%, respectively) (Fig. 3, A to F). Spatial heterogeneity was also observed within an organ (Fig. 3G), with the highest disturbances in the microbiota found in the proximal large intestine (sampling position 11, PERMANOVA *P* = 0.022, *R*^2^ = 12.36% and PERMANOVA *P* = 0.009, *R*^2^ = 10.715% for 12 and 89 days after infection, respectively). Sustained disturbances in the large intestine microbiota reflect our findings for the large intestine metabolome, while the discrepancies between cecal microbiota and metabolome findings may reflect sustained luminal rather than tissue alterations. Overall, the lack of resolution of microbiota alterations in these sites correlates well with our observation of continued alterations of the fecal microbiota and metabolome through early and persistent experimental *T. cruzi* infection (*4*). In accordance with prior reports (*4*, *11*), no significant differences in microbial community richness were observed (fig. S4) between infected and uninfected samples at 12 and 89 days after infection. The effect size (*R*^2^) of infection on the overall microbiota composition was lower than for our tissue metabolomics analysis, suggesting a stronger impact of infection on the tissue metabolome than on the microbiota, although, in both cases, the proximal large intestine was one of the major sites of infection-associated perturbation. This may reflect segregation of the microbiome from the site of infection (*12*) so that only indirect effects can be observed. Furthermore, cage and batch effects were found to have a larger impact on microbiome composition, while metabolome analysis was more robust to these effects, as we previously reported (*4*).

### Role of acylcarnitines in CD tolerance

**Fig. 4:**
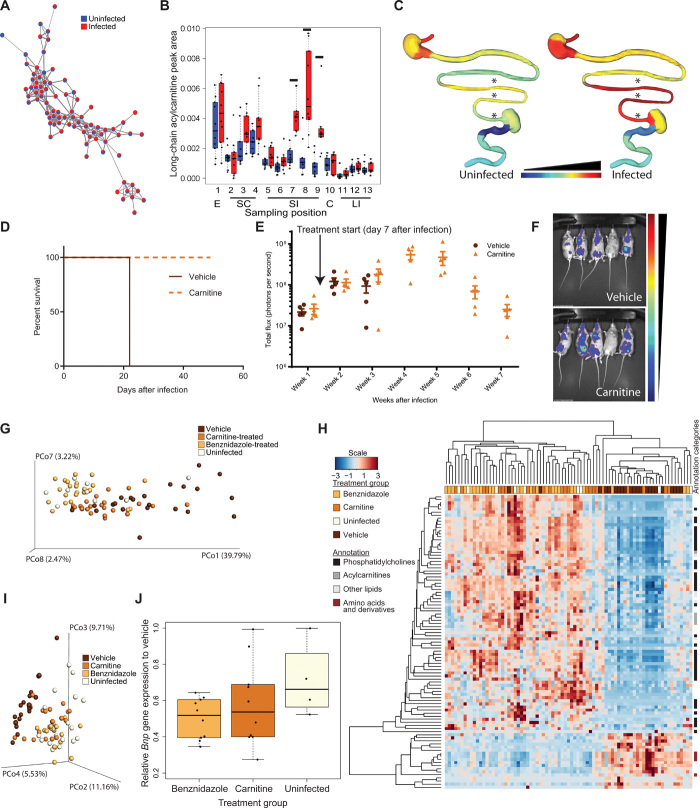
Chemical cartography reveals a causal role for carnitine metabolism in acute CD tolerance. (**A**) Acylcarnitine molecular network (all tissue sites and time points combined). (**B**) Infection-induced increases in acute-stage long-chain acylcarnitines in the distal small intestine (black lines, FDR-corrected Mann-Whitney *P* < 0.05). (**C**) Spatial distribution of acute-stage long-chain acylcarnitines [medians; common logarithmic scale from lowest (dark blue) to highest abundance (dark red)]. *Above sampling sites, *P* < 0.05 FDR-corrected Mann-Whitney. (**D**) Carnitine treatment prevents acute-stage mortality (*n* = 5 per group, 50,000 trypomastigote infection). (**E** and **F**) Comparable parasite burden between carnitine-treated and vehicle groups. (E) Overall whole-body luminescence. Mean and SEM are displayed. (F) Representative bioluminescent imaging, week 3 after infection (common scale). (**G** and **H**) Carnitine treatment mitigates plasma infection-induced metabolic disturbances (*n* = 24 benznidazole and carnitine groups, *n* = 22 vehicle group, and *n* = 9 uninfected group). (G) PCoA analysis (Bray-Curtis-Faith distance metric) of plasma samples. (H) Heat map showing metabolite features distinguishing vehicle-treated individuals from carnitine-treated, benznidazole-treated, and uninfected individuals (Kruskal-Wallis, FDR-corrected *P* < 0.05). (**I**) Carnitine treatment mitigates cardiac infection-induced metabolic disturbances. PCoA analysis (Bray-Curtis-Faith distance metric) of heart samples [*n* = 5 for uninfected, vehicle, and carnitine groups; *n* = 4 for benznidazole group (one biological replicate; see fig. S6D for data from second replicate)]. (**J**) Carnitine treatment reduces cardiac *Bnp* gene expression (*n* = 10 for benznidazole group and carnitine group, *n* = 9 for vehicle group, and *n* = 4 for uninfected group; Student’s *t* test *P* = 0.01).

Translating omics findings into novel therapeutic approaches is one of the major challenges of this post-genome era. Because we observed larger metabolome than microbiome infection-associated perturbations and on the basis of our current observations of infection-induced elevation in specific acylcarnitine family members and on our prior findings of differential cardiac acylcarnitine distribution and mass range in mild versus severe acute *T. cruzi* infection (*6*), we focused here on acylcarnitines and the potential of acylcarnitine modulation for CD treatment. The acylcarnitine subnetwork (Fig. 4A) was manually annotated (table S1), and impacts of infection on short-chain (C3-C4), mid-chain (C5-C11), and long-chain (C12 and greater) acylcarnitines were assessed. While total and short-chain GI acylcarnitine levels were comparable between infected and uninfected animals 12 days after infection (fig. S5, B and C), we observed significant elevation in total and short-chain acylcarnitine levels at each small intestine site in infected animals [total acylcarnitines: false discovery rate (FDR)–corrected Mann-Whitney *P* = 0.0054, *P* = 0.00042, *P* = 7 × 10^−5^, *P* = 7 × 10^−5^, and *P* = 0.00042 for positions 5, 6, 7, 8, and 9; short-chain acylcarnitines: FDR-corrected Mann-Whitney *P* = 0.00067, *P* = 0.00056, *P* = 0.00014, *P* = 0.00056, and *P* = 0.0019 for positions 5, 6, 7, 8, and 9; fig. S5, D and E]. For long-chain acylcarnitines, infection-induced acylcarnitine elevation was restricted to the distal portions of the small intestine (FDR-corrected Mann-Whitney *P* = 0.00014, *P* = 0.00056, and *P* = 0.00056 for positions 7, 8, and 9; Fig. 4, B and C). This difference between total and spatially resolved short-chain acylcarnitine levels highlights the strength of our chemical cartography approach. Mid-chain (C5 to C11) acylcarnitine levels were not significantly different 12 days after infection between infected and uninfected animals at any GI site (fig. S5F). Acylcarnitine small intestine elevation was no longer observed 89 days after infection, except for short-chain acylcarnitines in the duodenum (sampling position 5, FDR-corrected Mann-Whitney *P* = 0.025), although other select GI sites showed infection-induced increases in acylcarnitines (distal large intestine: total acylcarnitines and short-chain acylcarnitines, FDR-corrected Mann-Whitney *P* = 0.02 and *P* = 0.039, respectively; esophagus: short-chain acylcarnitines, FDR-corrected Mann-Whitney *P* = 0.0042; fig. S5, G to I). Acetylcarnitine was also elevated in select sites 12 days after infection [FDR-corrected Mann-Whitney *P* = 0.022, *P* = 0.0034, *P* = 0.00014, *P* = 0.00014, *P* = 0.00089, and *P* = 0.022 for site numbers 4 to 9 (stomach and small intestine, 12 days after infection)] but was comparable between infected and uninfected tissues at all sites 89 days after infection (fig. S5, J and K). In contrast, unmodified carnitine levels were comparable throughout the intestine 12 days after infection and only significantly elevated in infected esophagus and uninfected central large intestine 89 days after infection (FDR-corrected Mann-Whitney *P* = 0.00014 and *P* = 0. 019, respectively) (fig. S5L).

To determine whether we could translate these findings toward novel CD therapeutics and whether these acylcarnitine alterations play a causal role in disease progression, we assessed whether carnitine supplementation could alter acute CD outcome. Mice were infected with either a low dose (5000 trypomastigotes) or a high dose (50,000 trypomastigotes) of luciferase-expressing CL Brener parasites (*n* = 5 per group) (*3*). At 7 days after infection, animals were distributed into two groups of comparable parasite burden and one group switched from normal drinking water to drinking water supplemented with l-carnitine (equivalent to 100 mg/kg per day based on water consumption). Carnitine treatment completely abrogated acute CD-induced mortality up to 7 weeks after infection (*P* = 0.0027 Mantel-Cox test, 50,000 trypomastigote infection, Fig. 4D; 5000 trypomastigote infection, fig. S5M) but without any effect on parasite burden or parasite distribution (Fig. 4, E and F, and fig. S5N). This lack of antiparasitic activity is consistent with prior in vitro high-throughput screening data showing no impact of carnitine on parasite burden (*13*). Overall, these results indicate that acylcarnitine modulation by carnitine supplementation can induce disease tolerance in CD.

Given that metabolic adaptations represent major mechanisms of disease tolerance (*14*), to determine the mechanism of action of carnitine in acute CD survival, we first sought to determine the impact of carnitine supplementation on host metabolism. Plasma samples, samples from major CD sites (heart, esophagus, and large intestine), and samples from the small intestine [major site of infection-induced acylcarnitine perturbation in acute experimental CD; see Figs. 2 (A and C) and 4B] were collected from carnitine-treated, benznidazole-treated, vehicle control, and uninfected untreated animals and analyzed by LC-MS/MS. We observed that carnitine treatment “resets” host cardiovascular metabolism in infected animals by mitigating infection-induced metabolic perturbations in the plasma and the heart: Vehicle-treated animals present with a plasma and cardiac metabolite profile distinct from uninfected or benznidazole-treated animals, and this distance is reduced in carnitine-treated infected animals (plasma: Mann-Whitney *P* = 0.00059 for distance to benznidazole-treated animals and *P* = 0.004 for distance to uninfected animals; heart: Mann-Whitney *P* = 0.00043 for distance to benznidazole-treated animals and *P* = 0.00017 for distance to uninfected animals; Fig. 4, G and I, and fig. S6, A to D). In contrast, carnitine treatment had a much more minor impact on the esophagus and large intestine overall metabolite profile and did not restore metabolism in these tissues (Mann-Whitney *P* > 0.05 for pairwise comparisons between carnitine-treated and vehicle-treated animal distances to uninfected animals or to benznidazole-treated animals; fig. S6, E and F). The impact of carnitine treatment on the abundance of most individual heart and plasma metabolite features was, however, minor (Fig. 4H and fig. S6, H and I), suggesting that the restorative effect of carnitine treatment on cardiac and plasma metabolism results from the sum of multiple small changes on many metabolites rather than large alterations in select metabolites. Specific carnitine treatment–induced alterations include effects on plasma and heart lipids, including plasma PC family members, plasma acylcarnitines, and plasma amino acids (Kruskal-Wallis, FDR-corrected *P* < 0.05; Fig. 4H and fig. S6, H and I). Carnitine treatment also increased carnitine in the esophagus and small intestine (Mann-Whitney *P* = 0.0079 and *P* = 0.00037, respectively), acetylcarnitine in the small intestine (Mann-Whitney *P* = 0.00072), short-chain acylcarnitines in the small and large intestines (Mann-Whitney *P* = 0.0029 and *P* = 6.5 × 10^−6^, respectively), mid-chain acylcarnitines at the heart base and in the large intestine (Mann-Whitney *P* = 0.043 and *P* = 0.0014, respectively), long-chain acylcarnitines in the large intestine (Mann-Whitney *P* = 2.7 × 10^−5^), and total acylcarnitines in the small and large intestines (Mann-Whitney *P* = 0.018 and *P* = 2.7 × 10^−5^, respectively) (fig. S7).

In accordance with the lack of effect of carnitine treatment on parasite burden, no impact of carnitine treatment on cardiac inflammation (fig. S6J) or cardiac cytokine levels were observed (Student’s *t* test *P* > 0.05 comparing each quantified cardiac cytokine and chemokine between carnitine-treated and vehicle-treated animals; fig. S6K). As a proximal mechanism of its prosurvival effect, carnitine treatment significantly improved cardiac function: Cardiac brain natriuretic peptide (BNP) levels were decreased in carnitine-treated animals compared to vehicle-treated animals to levels comparable to those observed in benznidazole-treated and uninfected animals (Student’s *t* test *P* = 0.01 comparing carnitine-treated animals to vehicle-treated animals; Student’s *t* test *P* = 0.27 comparing carnitine-treated animals to uninfected animals; and Student’s *t* test *P* = 0.5 comparing carnitine-treated animals to benznidazole-treated animals; Fig. 4J). BNP is a biomarker of cardiac strain that is expressed at higher levels in the failing heart; reduction in BNP, as observed in carnitine-treated animals, indicates reduced myocardial wall stress (*15*). While carnitine can present antioxidant effects (*16*) and antioxidants reduce parasite load (*17*), given the lack of impact of carnitine treatment on parasite load, we consider it unlikely for carnitine’s main effect on CD progression to be via antioxidant mechanisms. Quantifying cardiac oxidized glutathione, a marker of oxidative stress (*18*), confirmed that carnitine treatment had limited impact on the oxidative environment in the heart (Mann-Whitney *P* = 0.558 comparing carnitine-treated and vehicle-treated groups; fig. S6L). As expected, oxidized glutathione levels were lower in uninfected mice than in infected animals (Mann-Whitney *P* = 0.02062 comparing infected vehicle-treated and uninfected animals; fig. S6L).

Overall, these findings have important implications for our understanding of the factors that contribute to the progression from asymptomatic to symptomatic CD. Our results further represent a novel avenue for CD drug development in conjunction with antiparasitics to kill *T. cruzi.*

## DISCUSSION

Disease severity is tied to the balance between resistance and tolerance mechanisms (*14*). Resistance reduces pathogen load but can cause collateral damage to the host, as has been observed with immune clearance of *T. cruzi*–infected cells (*1*). In contrast, tolerance reduces disease or immune-mediated collateral damage without affecting the pathogen load (*14*). While parasite persistence is required for progression to chronic CD, only a minority of infected patients progress to symptomatic disease, and parasite load does not fully predict disease severity (*1*). Our results demonstrating that carnitine modulation prevented acute-stage mortality without altering parasite load (Fig. 4 and fig. S5) provide further evidence for the importance of disease tolerance mechanisms in CD. These findings also represent the first time that carnitine metabolism has been directly linked to disease tolerance mechanisms in infection rather than serving as a readout for altered fatty acid metabolism. We determined the mechanism by which carnitine supplementation induces disease tolerance in CD by demonstrating that it mitigates infection-associated host metabolic dysfunction in the circulatory system. Acute *T. cruzi* infection significantly perturbs metabolism across the GI tract and circulatory system (Figs. 1 and 4, G and I). Carnitine supplementation reduced these perturbations in the heart and the plasma (Fig. 4, G and I) so that infected carnitine-treated animals become metabolically more similar to uninfected or benznidazole-treated animals and more dissimilar to infected vehicle-treated controls. Pathologic metabolic alterations are known drivers of noninfectious heart failure, and their correction through metabolic modulators reduce cardiac stress and improve heart function (*19*). We observed a similar mechanism for carnitine supplementation in acute CD, whereby carnitine improved cardiac metabolism, leading to reduced cardiac stress (as evidenced by reduced *Bnp* gene expression; Fig. 4J) and thus preventing acute-stage animal mortality. Overall, these findings pave the way for future studies of the role of acylcarnitines in the progression from asymptomatic to symptomatic CD in humans, as well as the development of new interventional strategies for CD, most likely in combination with antiparasitic agents to ensure complete patient cure and following additional safety and efficacy evaluation.

Notably, sites of largest statistically significant and increased overall metabolic disturbance in persistent infection were the esophagus and large intestine (Fig. 1J), providing a mechanism whereby sustained metabolic alterations at these sites drive the marked selective tropism of CD for the large intestine and esophagus, even when parasite load is low. Select PC family metabolites remained increased in the esophagus and large intestine in persistent infection (Fig. 2, D to F). Given the proinflammatory role of LPCs (*8*), this localized elevation in PCs may be contributing to the cycles of inflammatory tissue damage that drive CD. In contrast, parasite persistence 89 days after infection in the cecum was metabolically silent (Fig. 1, H and J), while cecal microbiota remained strongly and significantly affected by infection (Fig. 3E). It is tempting to speculate a microbiota-mediated mechanism of reduced antiparasitic immune responses in the cecum, perhaps via cecal microbiota-derived short-chain fatty acid (*20*) or induction of parasite dormancy at this site (*21*), leading to the observed parasite recrudescence in the cecum following incomplete antiparasitic treatment [e.g., (*2*)]. This awaits further experimentation. In this context, our spatial map of infection-induced metabolic changes can serve as a reference to begin identifying host and microbiota metabolic signals that could be modulating parasite dormancy.

One limitation of this study is that our methods are not able to distinguish between host-derived, microbiota-derived, and *T. cruzi–*derived metabolites. However, given the relative abundance of host and microbiota versus parasite metabolites, we anticipate most of the detected metabolites to be host or microbiota derived rather than parasite derived. Further investigation will also be required to determine whether these host metabolite changes are common across infection conditions or influenced by *T. cruzi* virulence. While some metabolite changes will likely be unique to a given mouse strain and parasite strain combination, mechanisms of CD pathogenesis, including parasite-mediated host cell lysis, immune-mediated cardiac damage, and subsequent cardiac fibrosis, are common across experimental systems and in humans (*1*). Thus, we anticipate many of the metabolic alterations induced by infection in our experimental system to be conserved. Many of the changes observed in this study, including increases in long-chain acylcarnitines in multiple sites, in kynurenine in the stomach and large intestine, and in select PCs in the esophagus and large intestine also match data obtained from heart tissue and plasma by Girones *et al.* (*22*) in BALB/c mice acutely infected with strain Y parasites. Our observed decrease in plasma acylcarnitines (remediated by carnitine treatment; Fig. 4H) also matches with recent findings that infection decreases many acylcarnitines in chronic CD (*23*). Likewise, several of our microbiome observations in this C3H/HeJ mouse model of infection with luminescent CL Brener parasites [this study and (*4*)] were corroborated in a study of *T. cruzi–*infected children (*11*), including the following: (i) absence of microbial richness changes associated with infection, (ii) infection-associated changes in community composition (beta diversity), and (iii) alterations in family Lachnospiraceae in response to infection.

We observed within-organ spatial heterogeneity in metabolite profile (Fig. 1D) and in response to infection at the microbiome and metabolome levels (Figs. 1J, 2, and 3G). Acylcarnitines, in particular, showed strong spatial effects that would have been masked by bulk tissue analysis (Figs. 2, A to C, and 4, B and C, and fig. S5). Systemic administration of carnitine nevertheless led to localized effects, demonstrating the strength and translatability of our spatially resolved approach into novel therapeutics. Likewise, the protective effects of carnitine supplementation in CD were missed by prior in vitro high-throughput screens (*13*). In vitro treatment with carnitine altered the cellular metabolite profile but did not restore in vitro infection-induced metabolic changes (fig. S6G), indicating that the protective effect of carnitine in CD requires the complex in vivo environment. These observations highlight the need for a spatial perspective in the study of host-microbe interactions and challenge existing approaches that treat anatomically homogeneous tissues as functionally homogenous. They also highlight the importance of considering in vivo rather than in vitro host-pathogen metabolic interactions, in a spatial context, for both rational drug target discovery and for studies of drug mechanism of action. The spatially resolved metabolomic and microbiome methods that we illustrate here in experimental *T. cruzi* infection can readily be applied to study other pathogens with specific tissue tropism, and we anticipate this approach to have broad applicability. Likewise, initial pathogen tropism is affected by tissue characteristics. Our comprehensive spatial characterization of the microbiome and metabolome of uninfected mice therefore represents a resource that can serve as a hypothesis-generating starting point for studies of pathogen tropism.

## MATERIALS AND METHODS

### Experimental design

#### Sample size

No power analyses were performed to predetermine sample size. Numbers were selected on the basis of our prior studies showing sufficient statistical power achieved with five mice per group (*6*).

#### Rules for stopping data collection

Time points for chemical cartography and microbiome analysis were predetermined at 12 and 89 days after infection to cover early and persistent *T. cruzi* infection. These time points also enabled comparison with our prior analysis of infected cardiac tissues, where samples were also collected 12 and 90 days after infection (*6*), and our prior studies of the microbiota in experimental CD, which terminated 3 months after infection (*4*). Prior time course analysis had also determined that significant disruptions of the overall fecal microbiome and metabolome emerge between 10 and 14 days after infection (*4*). End point for vehicle-treated mice in carnitine efficacy studies was based on humane weight loss end points. End point for carnitine treatment mechanism of action studies was predetermined to enable sample collection before expected time of death of vehicle-treated group in this model.

#### Data inclusion/exclusion criteria

No inclusion/exclusion criteria were applied in terms of animal selection. Samples that showed metabolite profile comparable to blanks by PCoA were excluded from subsequent analyses. Sequencing data were rarefied to a depth of 5000 reads per individual, established after comparing read numbers between blanks and samples.

#### Outliers

Samples that showed metabolite profile comparable to blanks by PCoA were excluded from subsequent analyses. Sequencing samples that showed reads <5000 were excluded from the study.

#### Selection of end points

End points were prospectively determined as described above, except for carnitine efficacy studies where humane animal weight loss cutoffs (predetermined, weight loss exceeding 20% compared to controls) were used.

#### Replicates

Animal experimentation was performed in biological duplicate. All collected GI samples were analyzed in one LC-MS run and in one sequencing run. Collected organ and plasma samples from carnitine treatment mechanism of action experiments were analyzed in one LC-MS run per organ. C2C12 treatment was performed in four biological replicates, which were analyzed in a single LC-MS run.

#### Research objectives

Research objectives were as follows: (i) to determine the spatial impact of *T. cruzi* infection on the microbiome and metabolome (predetermined objective), (ii) to determine whether carnitine modulation affects infection severity once this analysis revealed infection-associated changes in acylcarnitine profile, and (iii) to determine the protolerance mechanism of action of carnitine once carnitine treatment showed efficacy in acute disease.

#### Research subjects or units of investigation

Research subjects are rodents (male C3H/HeJ mice).

#### Experimental design

Results are derived from controlled laboratory experiments. Male C3H/HeJ mice were used for the in vivo studies. The chemical cartography and 16*S* analyses included (i) uninfected mice and (ii) infected mice. The carnitine mechanism of action experiments included the following four groups: (i) uninfected, (ii) infected and treated with water (vehicle group), (iii) infected and treated with benznidazole, and (iv) infected and treated with carnitine. Animal weight, survival, and parasite bioluminescence were quantified. Tissues and plasma were collected, and microbiome and metabolome composition was determined.

#### Randomization

Animals were initially randomly assigned to either be infected or not, on a per-cage basis. For carnitine efficacy experiments, 7 days after infection, animals were assigned to either vehicle or carnitine group by ensuring that average parasite burden (determined by average bioluminescence) was comparable between groups. LC-MS run order was random.

#### Blinding

No blinding was performed.

### In vivo experimentation

All vertebrate animal studies were performed in accordance with the U.S. Department of Agriculture Animal Welfare Act, the *Guide for the Care and Use of Laboratory Animals* of the National Institutes of Health, and all institutional guidelines in specific pathogen–free facilities. The protocols were approved by the University of California San Diego Institutional Animal Care and Use Committee (protocol S14187) and the University of Oklahoma Institutional Animal Care and Use Committee (protocol R17-035).

For chemical cartography and 16*S* analysis, 5-week-old male C3H/HeJ mice (the Jackson laboratory) were infected by intraperitoneal injection of 1000 red-shifted luciferase-expressing *T. cruzi* strain CL Brener (*3*) culture-derived trypomastigotes in 100 μl of Dulbecco’s modified Eagle’s medium (DMEM) (infected group) or mock-infected by injection of 100 μl of DMEM only (uninfected group). Before animal infection, *T. cruzi* parasites were maintained in coculture with C2C12 mouse myoblasts, in DMEM (Invitrogen) supplemented by 5% iron-supplemented calf serum (HyClone) and 1% penicillin-streptomycin (Invitrogen). Twelve or 89 days after infection, animals were injected with d-luciferin potassium salt (150 mg/kg; Gold Biotechnology) and euthanized by isoflurane overdose followed by cervical dislocation. Mice were then immediately perfused with 10 ml of ice-cold d-luciferin (0.3 mg/ml) in phosphate-buffered saline (PBS) (*3*) to remove circulating parasites, provide luciferase substrate, and prevent quenching of luminescent signal by hemoglobin (*24*). GI organs were collected and sectioned as displayed on Fig. 1B, and each section was placed in an individual 96-well plate containing ice-cold d-luciferin (0.3 mg/ml) in PBS (*3*). The plate was imaged in an In vivo Imaging System (IVIS) Lumina LT Series III (PerkinElmer), and tissues were then immediately snap-frozen in liquid nitrogen, followed by storage at −80°C. Tissue section luminescence was determined using Living Image 4.5 software, normalized to average of uninfected tissue luminescence collected at the same time point (replacing negative values with zero) and to tissue weight, and plotted in R. Comparisons of luminescent signal between groups were performed using a Kruskal-Wallis test with Dunn’s post hoc multiple comparison test, with Bonferroni correction, using R package “FSA.” The code is freely available on GitHub (see “Data and materials availability” section). Tissue luminescence is linearly related to parasite load, with a 10^7^-fold difference in luminescence between infected and uninfected tissues ex vivo corresponding to ca. 10^4^ parasite equivalents to 50 ng of murine DNA and a 10^3^-fold difference in luminescence between infected and uninfected tissues corresponding to ca. 1 parasite equivalents to 50 ng of murine DNA (*3*). Two biological replicate experiments were performed, each including *n* = 5 mice for each time point and infection condition (total *n* = 10 per time point and infection condition). The same samples were used as source material for 16*S* and LC-MS analysis (see below), with each tissue site from each individual animal representing a single data point in each analysis.

For carnitine supplementation efficacy experiments, mice were infected with either a low dose (5000 culture-derived trypomastigotes) or a high dose (50,000 culture-derived trypomastigotes) of red-shifted luciferase-expressing CL Brener parasites (*3*). Seven days after infection, mice were injected with d-luciferin potassium salt (150 mg/kg; Gold Biotechnology) and imaged (IVIS Lumina LT Series III). Animals were allocated to treatment groups to have comparable total body luminescence signal between groups. Mice then received l-carnitine (VWR) in drinking water ad libitum, normalized to mouse water consumptions so that animals received ca. 100 mg/kg per day, or regular drinking water (*n* = 5 per group). Bioluminescent imaging was performed weekly. Animals reaching humane end points of weight loss >20% were euthanized. Bioluminescence data were analyzed with Living Image 4.5 software and plotted using GraphPad Prism version 8.

For carnitine supplementation mechanism of action experiments, mice were infected with 50,000 culture-derived trypomastigotes of red-shifted luciferase-expressing CL Brener parasites (*3*). One group of mice was mock-injected with DMEM only (uninfected control group). Seven days after infection, mice received benznidazole (100 mg/kg per day by intraperitoneal injection in 10% dimethyl sulfoxide vehicle); l-carnitine (VWR) in drinking water ad libitum, normalized to mouse water consumptions so that animals received ca. 100 mg/kg per day; or regular drinking water (two biological replicates for treatment groups). Seventeen days after infection, animals were euthanized and plasma, heart [sectioned as in (*6*)], esophagus, small intestine, and large intestine collected and sectioned as in Fig. 1B. Tissues were either snap-frozen and then stored at −80°C (for LC-MS/MS), placed in RNAlater (Sigma-Aldrich) and then stored at −80°C (for gene expression analysis), or placed in 10% formalin at room temperature (VWR) and then paraffin-embedded, sectioned and hematoxylin and eosin–stained by the University of Oklahoma Health Sciences Center, Stephenson Cancer Center Tissue Pathology Shared Resource. Histological sections were digitized on a PathScan Enabler IV Histology Slide Scanner. For plasma metabolomics, *n* = 24 for benznidazole group and carnitine group, *n* = 22 for vehicle group (both biological replicates combined), and *n* = 9 for uninfected group. For tissue metabolomics, *n* = 9 for benznidazole group, *n* = 10 for carnitine group and vehicle group (both biological replicates combined), and *n* = 5 for uninfected group. For *Bnp* gene expression analysis by quantitative reverse transcriptase–polymerase chain reaction (qRT-PCR), *n* = 10 for benznidazole group and carnitine group, *n* = 9 for vehicle group (both biological replicates combined), and *n* = 4 for uninfected group. For fibrosis analysis by qRT-PCR, *n* = 5 for benznidazole group, carnitine group, and vehicle group and *n* = 4 for uninfected group (single experiment because no induction of fibrosis was observed in infected compared to uninfected animals). For histology, *n* = 5 for benznidazole group, carnitine group, and vehicle group (single experiment because no apparent difference was observed between carnitine-treated and vehicle-treated animals).

### Sample preparation for LC-MS/MS (tissue samples)

Samples from both biological replicate experiments were analyzed jointly. Metabolites were extracted from the collected tissue samples using a two-step process, as implemented in our prior work (*6*), normalizing to tissue weight. Tissue samples were homogenized in LC-MS–grade water (Fisher Optima): 50 mg of tissue in 125 μl of water (GI tract analysis, infected versus uninfected samples), 50 mg of tissue in 175 μl of water (carnitine treatment experiments: small intestine and large intestine), and 50 mg of tissue in 250 μl of water (carnitine treatment experiments: heart tissue and esophagus) using a 5-mm steel ball in Qiagen TissueLyser at 25 Hz for 3 min. Ten microliters was set aside for DNA extraction and microbiome profile analysis from GI samples, except for esophagus where the tissue amount was too small, and 100 μl was set aside for cytokine analysis (heart base only). LC-MS–grade methanol (Fisher Optima) spiked with 4 μM sulfachloropyridazine (Sigma-Aldrich; all tissue samples) and 2 μM heavy isotope–labeled lauroyl-l-carnitine-(*N*,*N*,*N*-trimethyl-d_9_) (Sigma-Aldrich; carnitine treatment samples) was added to the homogenized sample to a final concentration of 50% methanol, and the sample was homogenized again at 25 Hz for 3 min. Homogenate was centrifuged for 15 min at 14,980*g* at 4°C. The centrifugation supernatant was collected and dried in a Savant SPD111V (Thermo Fisher Scientific) SpeedVac concentrator. The centrifugation pellet was resuspended in 3:1 (by volume) dichloromethane (Fisher Optima)–to–methanol solvent mixture and further homogenized at 25 Hz for 5 min, followed by centrifugation at 14,980*g* for 2 min. This latter centrifugation supernatant was collected and air-dried. Both extracts were stored at −80°C until LC-MS analysis.

### Sample preparation for LC-MS/MS (plasma samples)

Samples from both replicates were analyzed jointly. Metabolite extraction was performed by adding 20 μl of plasma to 56 μl of 100% high-performance LC (HPLC)–grade methanol spiked with 0.5 μM sulfachloropyridazine (Sigma-Aldrich) and 0.5 μM heavy isotope–labeled lauroyl-l-carnitine-(*N*,*N*,*N*-trimethyl-d_9_; Sigma-Aldrich) to a final concentration of 74% methanol. The samples were vortexed for 15 s and centrifuged at 14,980*g* for 15 min. The supernatant was collected and dried in a Savant SPD111V (Thermo Fisher Scientific) SpeedVac concentrator overnight.

### Sample preparation for LC-MS/MS (cell culture samples)

C2C12 were infected at a 15:1 parasite–to–host cell ratio with luciferase-expressing *T. cruzi* strain CL Brener (*3*). Forty eight hours after infection, cells were treated with 80 μM carnitine or comparable volume of sterile water. Ninety-six hours after treatment, cultures were washed three times with ice-cold PBS, collected, snap-frozen, and stored at −80°C. Metabolites were extracted using 1:3:1 dichloromethane:methanol:H_2_O spiked with 2 μM sulfachloropyridazine (Sigma-Aldrich) and analyzed by LC-MS/MS (four independent biological replicates analyzed in a single LC-MS/MS run; *n* = 4 per infection and treatment groups).

### Liquid chromatography–tandem mass spectrometry

Dried tissue samples were resuspended in 50% methanol (Fisher Optima; LC-MS grade) spiked with 2 μM sulfadimethoxine (Sigma-Aldrich) as internal control (except for heart samples, which were resuspended in 50% methanol), pooling aqueous and organic extracts together. Dried plasma samples were resuspended in 150 μl of HPLC-grade water spiked with 0.5 μM sulfadimethoxine as internal control. LC was performed using a Thermo Scientific Vanquish UHPLC system fitted with 1.7 μm 100 Å Kinetex C8 column (50 × 2.1 mm) (Phenomenex; GI tract and heart) or 1.6 μm 100 Å Luna Omega Polar C18 column (50 × 2.1 mm) (Phenomenex; plasma samples and cell-culture samples), using water with 0.1% formic acid as mobile phase A and acetonitrile with 0.1% formic acid as mobile phase B. Data-dependent MS/MS experiments were performed on a Q Exactive Plus (Thermo Scientific) high-resolution MS, under the control of Xcalibur and Tune software (Thermo Scientific). Ions were generated for MS/MS analysis in both positive and negative ion mode using heated electrospray ionization source. Calibration of the instrument was performed using recommended commercial calmix from Thermo Scientific. See table S2 for detailed instrumental parameters.

### LC-MS/MS data analysis

Raw data were converted to mzXML format using MSConvert software (*25*). Processing of the resulting mzXML files was done in MZmine versions 2.30 (GI tract), 2.33 (carnitine treatment tissue samples, except small intestine), 2.37 (carnitine treatment C2C12 samples), and 2.51 (carnitine treatment small intestine samples) (see table S2 for parameters) (*26*). Data were filtered to only retain MS1 scans that were present in at least six samples (GI tract) or four samples (carnitine treatment samples) and were associated with MS2 spectra (and therefore could potentially be annotated). Blank removal was performed, with a minimum threefold difference between blank and samples required for a feature to be retained. Total ion current (TIC) normalization was performed in Jupyter Notebook using R (http://jupyter.org). PCoA was performed on the TIC-normalized MS1 data using the Bray-Curtis-Faith dissimilarity metric in QIIME 1 (*27*), visualized using EMPeror (*28*). PERMANOVA calculations were performed on Bray-Curtis-Faith distance matrices using the R package “vegan” (*29*). Samples close together in PCoA plot space indicate samples with similar metabolite profiles. Higher PERMANOVA *R*^2^ values indicate a greater relationship between the chemical variation and the selected metadata variable. Between-group average distance calculations were performed on the Bray-Curtis-Faith dissimilarity matrix using categorized_dist_scatterplot.py in QIIME 1 (*27*). The GI tract model was built to scale from a picture of the GI tract collected from an additional uninfected C3H/HeJ mouse, using SketchUp 2017 software. Data were plotted onto this model using ‘ili (https://ili.embl.de/) (*30*). All ‘ili plots are scaled so that the lowest displayed abundance is assigned the coldest color and the highest abundance is assigned the warmest color. Individual scales are used for all plots, unless otherwise specified. Feature annotation was performed through molecular networking on the Global Natural Products Social Molecular Networking platform (*31*, *32*) with the following parameters: pairs and library search minimum cosine, 0.7; precursor ion and fragment ion mass tolerance, 0.02 Da; four minimum matched peaks, TopK, 10; MS cluster enabled and minimum cluster size (for standard molecular networking workflow only), 4; maximum component size, 100; minimum peak intensity, 0; filter below SD peak intensity, 0; filter precursor window and filter library; 50-Da window filter; maximal mass shift, 100 Da (GI tract) or 500 Da (carnitine treatment experiments); row sum normalization (feature-based molecular networking only); aggregation method per group, sum (plasma) or mean (carnitine treatment experiments, tissue samples); and top results per query, 1 (feature-based molecular networking only). All annotations are levels 2 or 3 according to the metabolomics standards initiative (*33*). Molecular networks were visualized using Cytoscape (*34*). Venn diagrams were generated using http://bioinformatics.psb.ugent.be/webtools/Venn/. Random forest classification (*35*) was performed in Jupyter Notebook using the randomForest R package and 7501 trees. To determine the metabolic impact of carnitine treatment on plasma and heart, Kruskal-Wallis tests were used to compare median feature counts as a function of treatment. FDR-adjusted *P* < 0.05 was used to identify features with significant associations with treatments. In addition, for each feature, pairwise Wilcoxon tests were run to identify the specific category/categories showing significant differences in medians. A Bonferroni-adjusted *P* < 0.05 was used to identify significant differences between each pair of treatments. A heat map was constructed using the “heat plot” function as implemented in the “made4” R package (*36*). Dendrograms are generated using unweighted pair group method with arithmetic mean clustering. Heat map colors depict normalized abundance determined from *Z* score calculations ([abundance − mean]/SD, between sample followed by within sample). Representative code can be accessed at https://github.com/mccall-lab-OU/GI-tract-paper.

### 16*S* method and data analysis

DNA was extracted from homogenized tissue samples using the DNeasy PowerSoil Kit (Qiagen) following the manufacturer’s protocols. Microbial load was determined using qPCR (FastStart Essential DNA Green kit, Roche) with primers (515F and 806R) targeting the 16*S* ribosomal RNA (rRNA) gene. Samples were normalized on the basis of microbial load, and the V4 hypervariable region of the 16*S* rRNA gene was amplified using barcoded Illumina-compatible primers 515F and 806R as previously described (*37*). The resulting amplicons were pooled in equimolar proportions and sequenced on an Illumina MiSeq Instrument. Paired-end sequencing reads were quality-filtered and merged to reconstruct the complete V4 region using AdapterRemoval version 2 (*38*). These analysis-ready reads were used to identify operational taxonomic units (OTUs) following the UNOISE pipeline implemented in USEARCH version 10 (*39*). Taxonomy was assigned to the representative OTUs using the EzTaxon database (*40*). The resulting OTU table was rarefied to a depth of 5000 reads per individual, and all downstream statistical analyses were performed using this rarefied OTU table. Analyses of microbial community richness (alpha diversity quantified as observed species) and community composition [beta diversity quantified as unweighted UniFrac (*41*)] analyses were performed using scripts implemented in QIIME 1 (*27*). Kruskal-Wallis tests with FDR correction were used for comparison of genus-level taxonomic summaries to infection status and disease stage.

### Quantitative RT-PCR

RNAlater-preserved tissue from the heart base was homogenized for 6 min with 5-mm stainless steel beads in DNA/RNA shield (Zymo Research) and RNA extracted using the Quick-DNA/RNA MiniPrep Plus Kit (Zymo Research). mRNA was reverse-transcribed into cDNA (Invitrogen High-Capacity cDNA Reverse Transcription Kit with RNase Inhibitor), and the cDNA was used for qRT-PCR using the following primers: GACTTCAACAGCAACTCCCAC and TCCACCACCCTGTTGCTGTA (*Gapdh*), CCTGGTAAAGATGGTGCC and CACCAGGTTCACCTTTCGCACC (collagen I), and AAGTCCTAGCCAGTCTCCAGA and GAGCTGTCTCTGGGCCATTTC (*Bnp*) (*42*). Changes in gene expression were analyzed using the ΔΔ*C*T method.

### Cardiac cytokine analysis

Heart base homogenate was analyzed using the Q-Plex Mouse Cytokine Screen (16-plex; Quansys Biosciences), according to manufacturers’ protocol and as previously described (*6*). Data were processed using Q-View software. Values below the limit of detection (LOD) of the kit for each cytokine were replaced with the LOD.

### Statistical analysis

All statistical tests are paired. Nonparametric tests were used where possible (Mann-Whitney *U* test and Kruskal-Wallis test with Dunn’s post hoc test with Bonferroni correction), which make no assumptions as to data normality. No additional tests of normality were performed. For acylcarnitine data analysis and Fig. 2, where Mann-Whitney *U* tests were performed for each sampling site, FDR correction was performed to adjust for multiple comparison, as specified in the text and in the figure legends. Box plots display first quartile, median, and third quartile, with whiskers no more than 1.5 times the interquartile range.

## Supplementary Material

aaz2015_Data_file_S1.xlsx

aaz2015_SM.pdf

## SUPPLEMENTARY MATERIALS

Supplementary material for this article is available at http://advances.sciencemag.org/cgi/content/full/6/30/eaaz2015/DC1

## Appendix

View/request a protocol for this paper from *Bio-protocol*.
