# Supplementary material for: Mapping of host-parasite-microbiome interactions reveals metabolic determinants of tropism and tolerance in Chagas disease
Source: Sci Adv. 2020 Jul 22;6(30):eaaz2015. doi: 10.1126/sciadv.aaz2015 (PMC7385396; doi:10.1126/sciadv.aaz2015)
Supplement: aaz2015_SM.pdf [file aaz2015_SM.pdf]

Science Advances
AAAS

advances.sciencemag.org/cgi/content/full/6/30/eaaz2015/DC1

Supplementary Materials for

Mapping of host-parasite-microbiome interactions reveals metabolic determinants of tropism and tolerance in Chagas disease

E. Hossain, S. Khanam, D. A. Dean, C. Wu, S. Lostracco-Johnson, D. Thomas, S. S. Kane, A. R. Parab, K. Flores, M. Katemauswa, C. Gosmanov, S. E. Hayes, Y. Zhang, D. Li, C. Woelfel-Monsivais, K. Sankaranarayanan*, L.-I. McCall*

*Corresponding author. Email: ksankar@ou.edu (K.S.); lmccall@ou.edu (L.-I.M.)

Published 22 July 2020, *Sci. Adv.* **6**, eaaz2015 (2020)
DOI: 10.1126/sciadv.aaz2015

The PDF file includes:

Figs. S1 to S7
Tables S1 and S2
Legend for data file S1

Other Supplementary Material for this manuscript includes the following:

(available at advances.sciencemag.org/cgi/content/full/6/30/eaaz2015/DC1)

Data file S1

Supplementary Materials

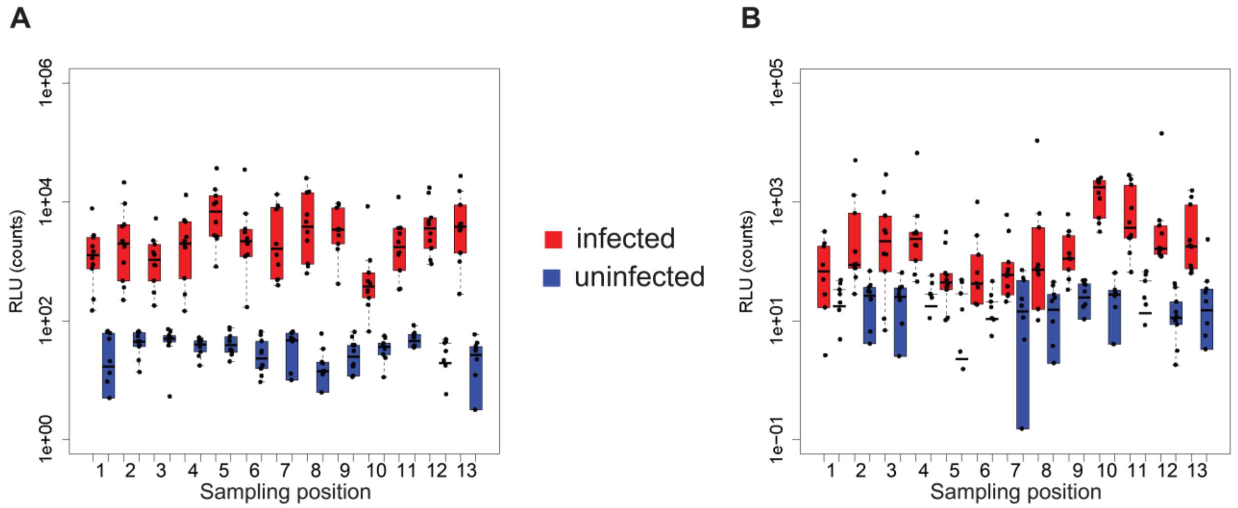

Fig. S1. Overall higher luminescence, representing parasite burden, in infected samples than background luminescence from uninfected samples. (**A**) 12 days post-infection. (**B**) 89 days post-infection. RLU, relative luminescence units. Note that this data is not adjusted for section size (weight). For normalized data, refer to **Fig. 1A**.

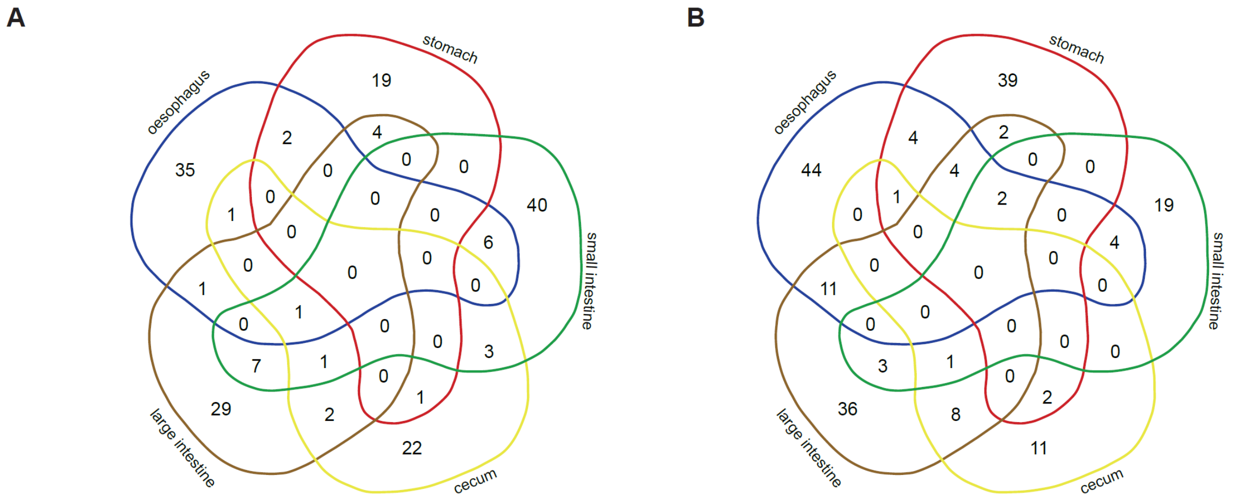

Fig. S2. Limited overlap in chemical families differentially-modulated by infection, across organs. Metabolite features were combined into chemical families (as identified by feature-based molecular networking (*31*)). Chemical families with fold change in abundance >2 and Mann-Whitney p-value<0.05 were compared between sampling sites. (**A**) 12 days post-infection. (**B**) 89 days post-infection.

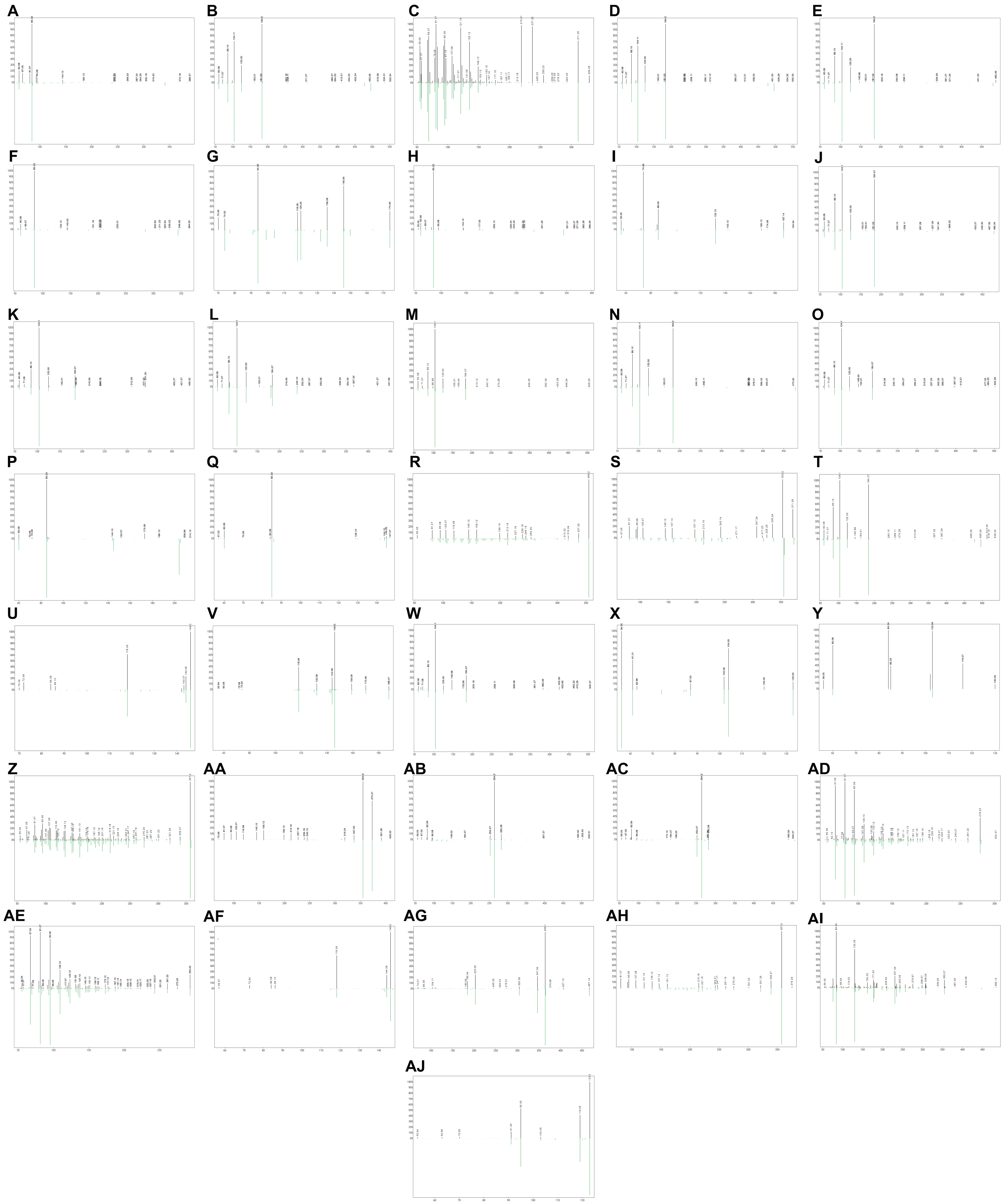
A
B
C
D
E
F
G
H
I
J
K
L
M
N
O
P
Q
R
S
T
U
V
W
X
Y
Z
AA
AB
AC
AD
AE
AF
AG
AH
AI
AJ

Fig. S3. Annotation support for infection-modulated molecules identified by random forest analysis and for microbially-derived and microbially-modified molecules. All annotations are level 2/3 according to the metabolomics standards initiative (*33*). Top (black), experimental MS2 spectrum. Bottom (green), library reference. (**A**) *m/z* 448.341 RT 2.90 min match to palmitoylcarnitine. (**B**) *m/z* 570.355 RT 2.96 min match to LPC(16:0). (**C**) *m/z* 329.268 RT 3.02 min match to monopalmitolein. (**D**) *m/z* 568.339 RT 2.90 min match to LPC(16:0). (**E**) *m/z* 544.339 RT 2.90 min match to LPC(16:0). (**F**) *m/z* 388.305 RT 2.73 min match to lauryolcarnitine. (**G**) *m/z* 209.092 RT 0.63 min match to L-kynurenine. (**H**) *m/z* 442.352 RT 2.85 min match to lauryolcarnitine. (**I**) *m/z* 233.149 RT 0.82 min match to thr-leu. (**J**) *m/z* 510.391 RT 3.08 min match to LPC(16:0). (**K**) *m/z* 482.36 RT 2.97 min match to Lyso PAF C-18 (LPC(O-18:0)). (**L**) *m/z* 508.376 RT 3.00 min match to 1-(1Z-Hexadecenyl)-sn-glycero-3-phosphocholine. (**M**) *m/z* 564.437 RT 3.26 min match to LPC(O-18:0). (**N**) *m/z* 550.386 RT 3.08 min match to LPC(16:0). (**O**) *m/z* 538.422 RT 3.24 min match to Lyso-PAF C-18. (**P**) *m/z* 232.154 RT 1.00 min match to acetyl-DL-carnitine. (**Q**) *m/z* 204.123 RT 0.28 min match to acetyl-DL-carnitine. (**R**) *m/z* 373.273 RT 2.71 min match to cholic acid. (**S**) *m/z* 407.279 RT 2.68 min match to cholic acid. (**T**) *m/z* 572.37 RT 2.99 min match to LPC(16:0). (**U**) *m/z* 188.071 RT 1.19 min match to tryptophan. (**V**) *m/z* 205.097 RT 1.24 min match to tryptophan. (**W**) *m/z* 566.321 RT 2.91 min match to Lyso PAF C-18 (LPC(O-18:0)). (**X**) *m/z* 150.058 RT 0.38 min match to methionine. (**Y**) *m/z* 162.112 RT 0.26 min match to L-carnitine. (**Z**) *m/z* 357.278 RT 2.88 min match to chenodeoxycholic acid. (**AA**) *m/z* 817.518 RT 2.73 min match to cholic acid. (**AB**) *m/z* 538.519 RT 4.07 min match to Cer(d18:1/20:1). (**AC**) *m/z* 520.508 RT 4.06 min match to Cer(d18:1/16:1). (**AD**) *m/z* 279.231 RT 2.97 min match to 9(10)-EpOME. (**AE**) *m/z* 293.247 RT 3.15 min match to 9Z,11E,13E-octadecatrienoic acid ethyl ester. (**AF**) *m/z* 188.071 RT 2.18 min match to indole-l-lactate. (**AG**) *m/z* 527.158 RT 0.265 min match to maltotriose. (**AH**) *m/z* 785.589 RT 2.891 min

match to deoxycholic acid. (**AI**) *m/z* 307.083 RT 0.350 min match to oxidized glutathione (heart tissue, carnitine treatment). (**AJ**) *m/z* 165.055 RT 0.403 min match to tyrosine.

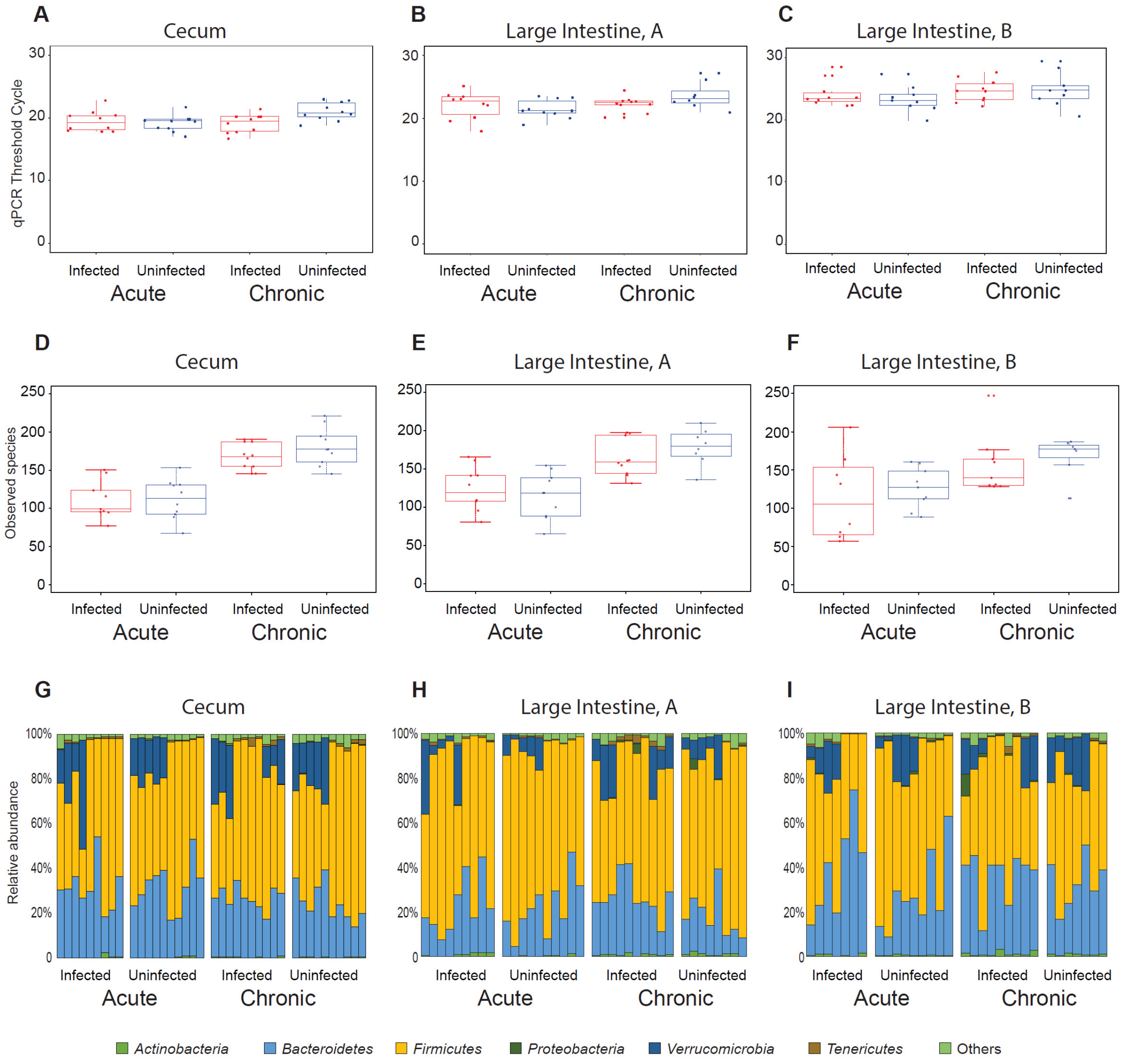

Fig S4. No impact of infection on microbial load and community richness. (**A-C**) Threshold cycle number for 16S rRNA gene quantitative PCR. (**D-F**) Microbial community richness, and (**G-I**) relative abundance of microbial phyla. Sampling sites: Cecum (position 10), proximal large intestine (position 11), and central large intestine (position 12).

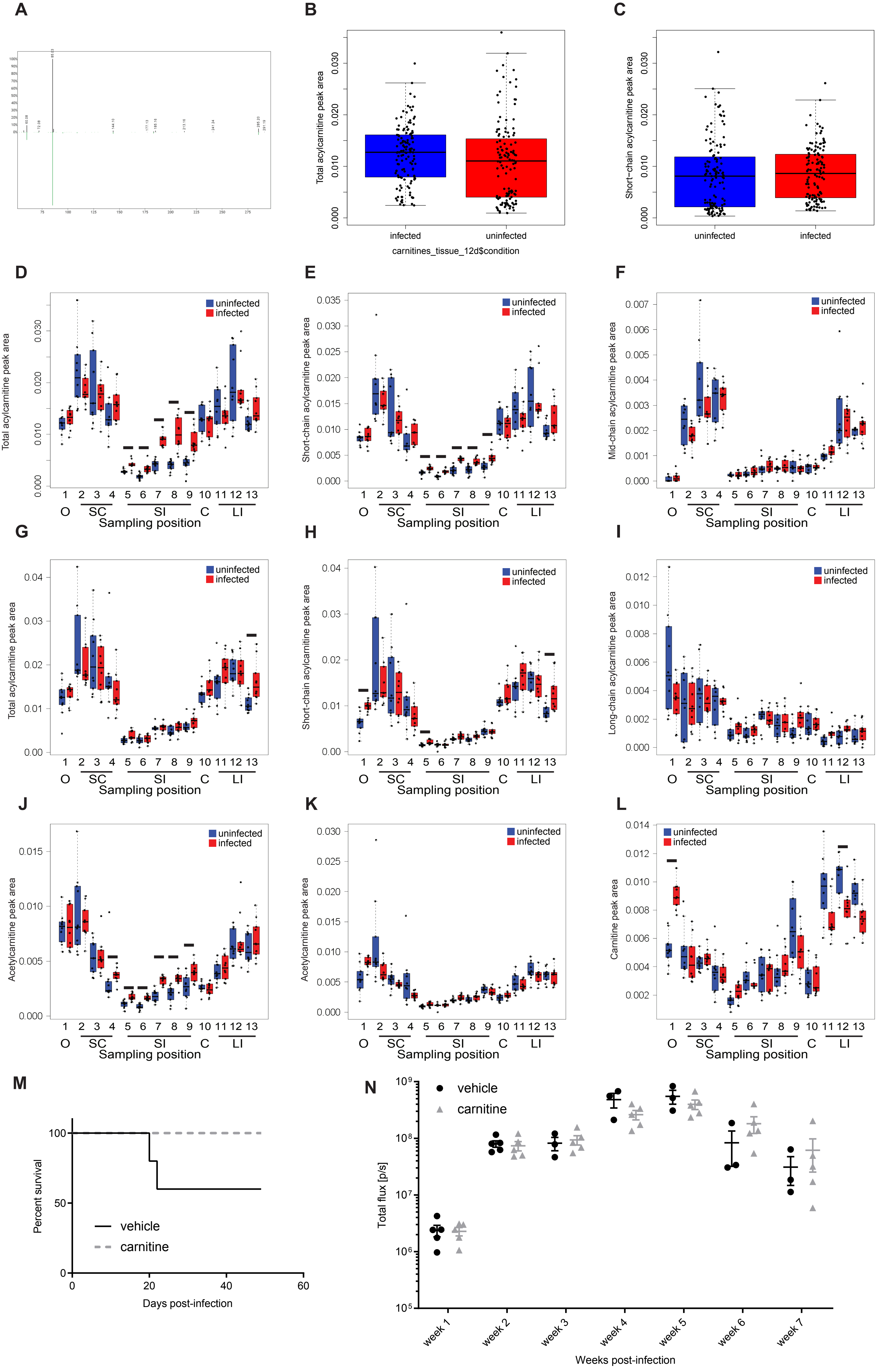
A
B
Total acylcarnitine peak area
infected
uninfected
carnitines_tissue_12d$condition
C
Short−chain acylcarnitine peak area
uninfected
infected
D
Total acylcarnitine peak area
uninfected
infected
1 2 3 4 5 6 7 8 9 10 11 12 13
O SC SI C LI
Sampling position
E
Short-chain acylcarnitine peak area
F
Mid-chain acylcarnitine peak area
G
Total acylcarnitine peak area
H
Short-chain acylcarnitine peak area
I
Long-chain acylcarnitine peak area
J
Acetylcarnitine peak area
K
Acetylcarnitine peak area
L
Carnitine peak area
M
Percent survival
Days post-infection
vehicle
carnitine
N
Total flux [p/s]
vehicle
carnitine
week 1
week 2
week 3
week 4
week 5
week 6
week 7
Weeks post-infection

Fig. S5. GI acylcarnitine abundance during infection and impact of carnitine supplementation on low-dose *T. cruzi* infection. (**A**) GNPS mirror plot showing MS2 match of *m/z* 344.279 RT 2.752 min to lauroylcarnitine library reference (green, bottom). (**B**) Total acylcarnitine peak area across all GI sites, 12 days post-infection. (**C**) Total short-chain acylcarnitine peak area across all GI sites, 12 days post-infection. (**D**) Total acylcarnitine peak area at each sampling site, 12 days post-infection. (**E**) Short-chain acylcarnitine peak area at each sampling site, 12 days post-infection. (**F**) Mid-chain acylcarnitine peak area at each sampling site, 12 days post-infection. (**G**) Total acylcarnitine peak area at each sampling site, 89 days post-infection. (**H**) Short-chain acylcarnitine peak area at each sampling site, 89 days post-infection. (**I**) Long-chain acylcarnitine peak area at each sampling site, 89 days post-infection. (**J**) Acetylcarnitine peak area at each sampling site, 12 days post-infection. (**K**) Acetylcarnitine peak area at each sampling site, 89 days post-infection. (**L**) Carnitine peak area at each sampling site, 89 days post-infection. Black bars, FDR-corrected Mann-Whitney $p<0.05$. (**M**) and (**N**) Male C3H/HeJ mice were infected with 5,000 luciferase-expressing *T. cruzi* strain CL Brener trypomastigotes. Beginning 7 days post-infection, animals received drinking water supplemented with carnitine (100 mg/kg/day equivalent, based on water consumption) or remained on standard drinking water (vehicle group). (M) Acute-stage humane endpoints and mortality were only observed in the vehicle-treated group. (N) Comparable total body parasite burden between groups, weeks 1-7 post-infection. Mean + standard error of mean displayed.

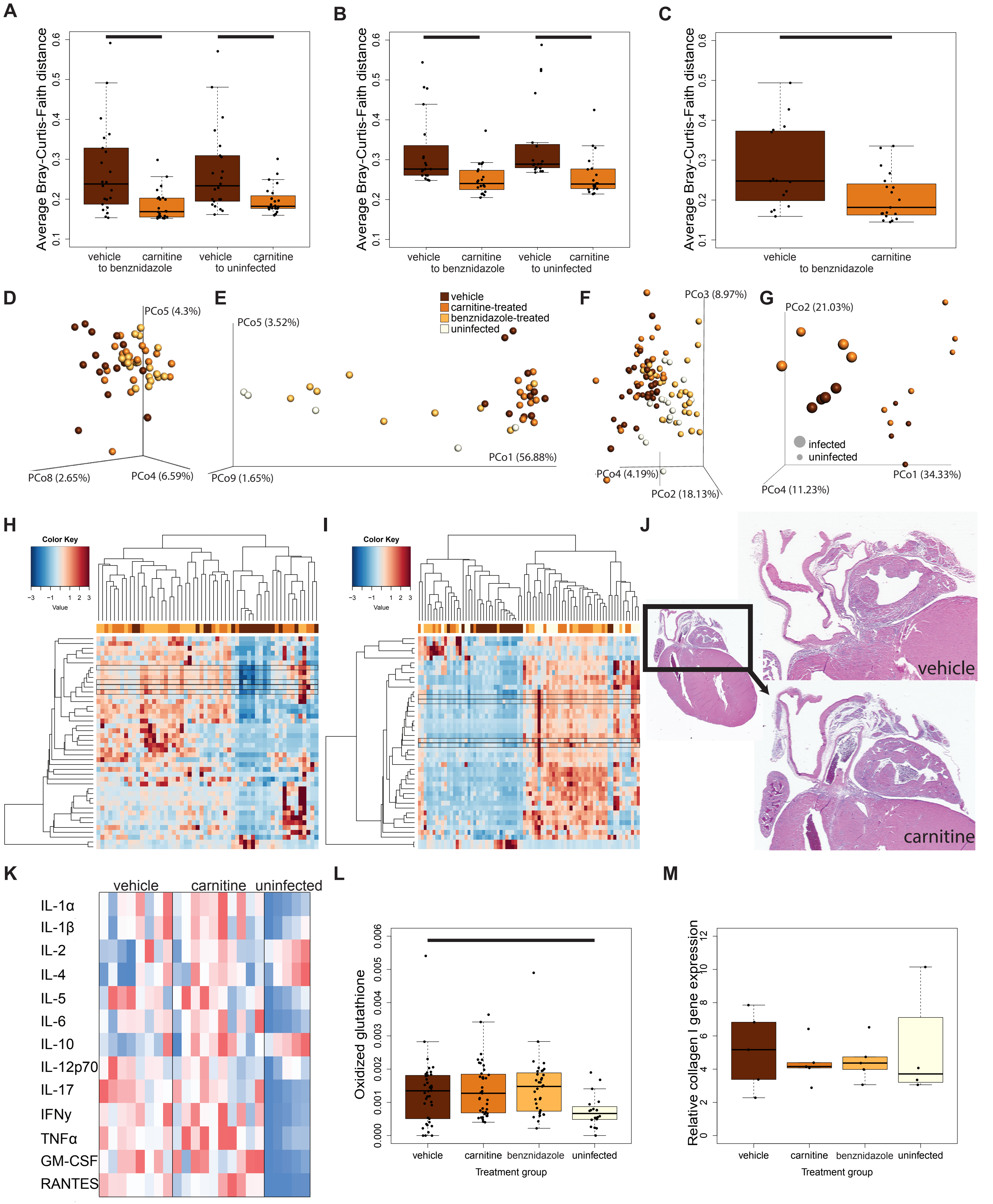
A
B
C
Average Bray-Curtis-Faith distance
vehicle
carnitine
to benznidazole
to uninfected
D
E
F
G
PCo5 (4.3%)
PCo8 (2.65%)
PCo4 (6.59%)
PCo5 (3.52%)
PCo9 (1.65%)
PCo1 (56.88%)
vehicle
carnitine-treated
benznidazole-treated
uninfected
PCo3 (8.97%)
PCo4 (4.19%)
PCo2 (18.13%)
PCo2 (21.03%)
PCo4 (11.23%)
PCo1 (34.33%)
infected
uninfected
H
I
J
Color Key
Value
vehicle
carnitine
K
L
M
IL-1α
IL-1β
IL-2
IL-4
IL-5
IL-6
IL-10
IL-12p70
IL-17
IFNγ
TNFα
GM-CSF
RANTES
Oxidized glutathione
Relative collagen I gene expression
benznidazole
uninfected
Treatment group

Fig. S6. Carnitine treatment resets cardiovascular metabolism in the absence of similar effects on GI tract metabolism and on cardiac inflammation. (**A**) Reduced Bray-Curtis-Faith distance between plasma metabolite profiles of carnitine and benznidazole-treated animals and carnitine and uninfected animals, compared to vehicle-treated and benznidazole-treated animals and vehicle-treated and uninfected animals. (**B**) and (**C**) Reduced Bray-Curtis-Faith distance between heart metabolite profiles of carnitine and benznidazole-treated animals and carnitine and uninfected animals, compared to vehicle-treated and benznidazole-treated animals and vehicle-treated and uninfected animals. (B) Replicate 1. (C) Replicate 2. (A), (B) and (C), Black lines, Mann-Whitney $p<0.05$. (**D**) PCoA analysis (Bray-Curtis-Faith distance metric) of heart samples, after 10 days of treatment (replicate 2). (**E**) and (**F**) Carnitine treatment does not mitigate infection-induced metabolic disturbances in the oesophagus (E) and the large intestine (F) after 10 days of treatment (both replicates combined). (**G**) Carnitine treatment alters metabolite profile but does not mitigate infection-induced metabolic disturbances in cell culture. PCoA analysis (Bray-Curtis-Faith distance metric) of *T. cruzi*-infected C2C12 cells, after 4 days of treatment (four replicates combined). (**H**) and (**I**) Heatmap showing cardiac metabolite features distinguishing vehicle-treated individuals from carnitine-treated and benznidazole-treated individuals (Kruskal-Wallis, FDR-corrected $p < 0.05$). (H) Replicate 1. (I) Replicate 2. Boxed: oxidized linoleic acid metabolites. (**J**) Comparable cellular infiltrate at the heart base between carnitine-treated and vehicle-treated animals. Representative H&E staining of histological sections at 7 days post-infection and 10 days post-treatment. Top, vehicle. Bottom, carnitine. (**K**) Comparable cardiac cytokine and chemokine profile between carnitine-treated and vehicle-treated animals (both replicates combined). (**L**) Carnitine treatment does not affect infection-induced increases in cardiac oxidized glutathione in infected animals (untargeted LC-MS/MS peak area, both replicates combined). Black line, Mann-Whitney $p<0.05$. (**M**) No significant induction of cardiac

fibrosis at 7 days post-infection and 10 days post-treatment (17 days post-infection). Collagen I ΔCt to *Gapdh* gene expression (single replicate).

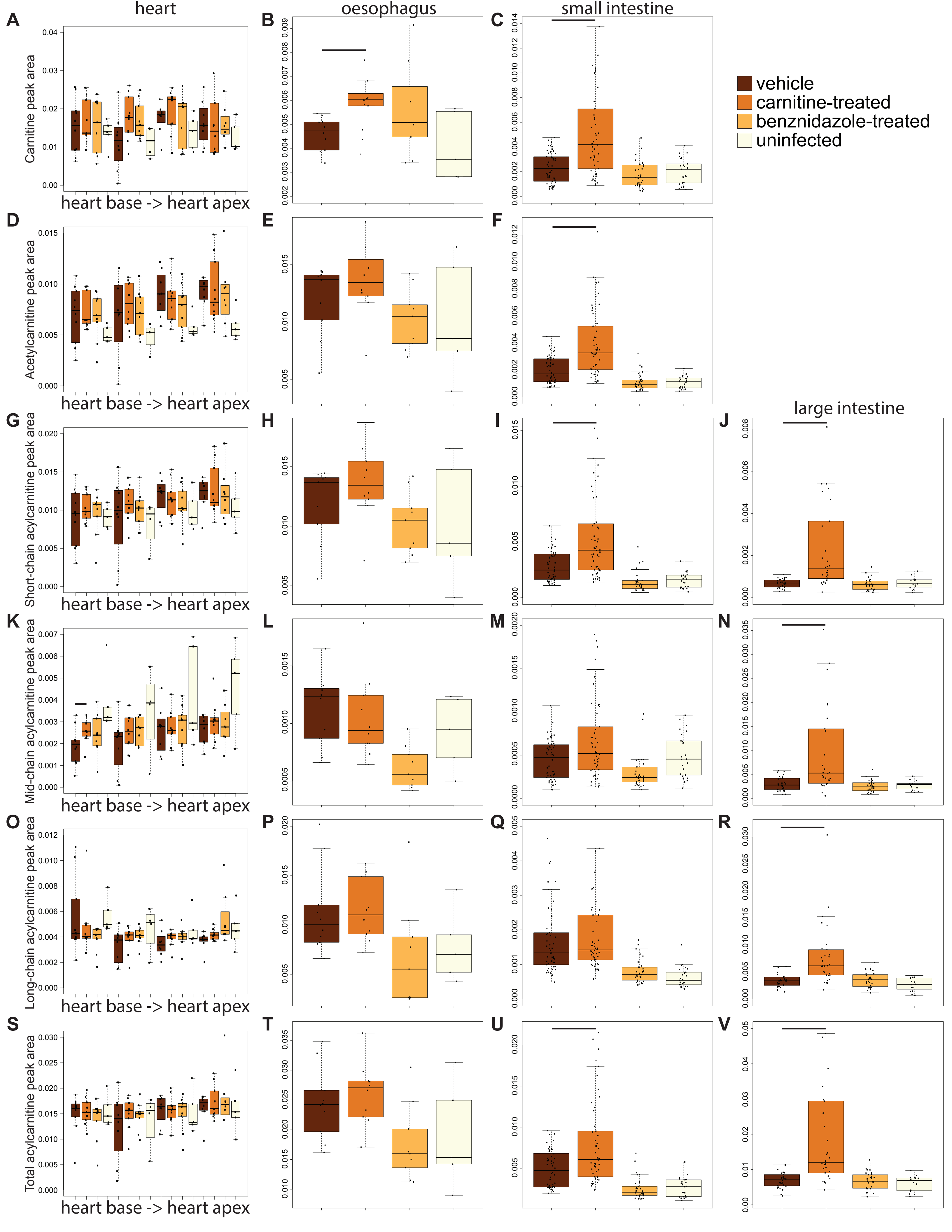
heart
oesophagus
small intestine
large intestine
vehicle
carnitine-treated
benznidazole-treated
uninfected
A
B
C
D
E
F
G
H
I
J
K
L
M
N
O
P
Q
R
S
T
U
V
Carnitine peak area
Acetylcarnitine peak area
Short-chain acylcarnitine peak area
Mid-chain acylcarnitine peak area
Long-chain acylcarnitine peak area
Total acylcarnitine peak area
heart base -> heart apex
heart base -> heart apex
heart base -> heart apex
heart base -> heart apex
heart base -> heart apex
heart base -> heart apex

Fig. S7. Impact of carnitine treatment on gastrointestinal and cardiac acylcarnitines. (**A-C**) Carnitine. (**D-F**) Acetylcarnitine. (**G-J**) Short-chain acylcarnitines. (**K-N**) Mid-chain acylcarnitines. (**O-R**) Long-chain acylcarnitines. (**S-V**) Total acylcarnitines. (A), (D), (G), (K), (O), (S) Heart. (B), (E), (H), (L), (P), (T) Oesophagus. (C), (F), (I), (M), (Q), (U) Small intestine. (J), (N), (R), (V) Large intestine. Carnitine and acetylcarnitine were not detected under our filtering criteria in the large intestine. Black lines, Mann-Whitney $p<0.05$.

Table S1. Detected acylcarnitines in GI tract samples.

Short-chain acylcarnitines			
m/z	**RT (min)**	**Putative annotation**	**ppm error**
204.123	0.282	C2:0 acylcarnitine (acetylcarnitine)	2.94
218.138	0.466	C3:0 acylcarnitine (propionylcarnitine)	5.50
232.154	0.349	C4:0 acylcarnitine (butyrylcarnitine)	0
232.154	1.002	C4:0 acylcarnitine (butyrylcarnitine)	0
248.149	0.404	C4:0-OH acylcarnitine	3.22
248.149	0.267	C4:0-OH acylcarnitine	3.22
**Mid-chain acylcarnitines**			
m/z	**RT (min)**	**Putative annotation**	**ppm error**
246.17	2.205	C5:0 acylcarnitine (valerylcarnitine)	2.03
260.185	2.304	C6:0 acylcarnitine	4.61
260.186	2.383	C6:0 acylcarnitine	0.77
288.216	2.514	C8:0 acylcarnitine	4.20
314.233	2.599	C10:1 acylcarnitine	0.32
316.247	2.645	C10:0 acylcarnitine	5.69
332.243	2.546	C10:0-OH acylcarnitine	2.11
**Long-chain acylcarnitines**			
m/z	**RT (min)**	**Putative annotation**	**ppm error**
342.263	2.865	C12:1 acylcarnitine	4.09
344.279	2.752	C12:0 acylcarnitine (lauroylcarnitine) [a]	3.20
360.273	2.655	C12:0-OH acylcarnitine	2.78
368.279	2.808	C14:2 acylcarnitine	2.99
370.294	2.789	C14:1 acylcarnitine	4.59
370.295	2.982	C14:1 acylcarnitine	1.89
372.237	2.399	C12:2-DC acylcarnitine	4.30
372.310	2.998	C14:0 acylcarnitine	3.76
372.310	2.835	C14:0 acylcarnitine	3.76
388.305	2.732	C14:0-OH acylcarnitine	1.80

396.310	2.829	C16:2 acylcarnitine	3.53
400.269	2.605	C14:2-DC acylcarnitine	2.25
400.341	2.929	C16:0 acylcarnitine (palmitoylcarnitine)	4.25
402.284	2.636	C14:1-DC acylcarnitine	3.98
412.305	2.72	C16:2-OH acylcarnitine	3.15
414.32	2.773	C16:1-OH acylcarnitine	4.59
414.321	2.841	C16:1-OH acylcarnitine	2.17
422.326	2.848	C18:3 acylcarnitine	2.37
424.341	2.896	C18:2 acylcarnitine	4.01
426.357	2.953	C18:1 acylcarnitine (oleoylcarnitine)	3.05
428.3	2.67	C16:2-DC acylcarnitine	2.80
428.373	3.018	C18:0 acylcarnitine	2.33
430.316	2.698	C16:1-DC acylcarnitine	2.09
440.336	2.823	C18:2-OH acylcarnitine	3.63
442.352	2.853	C18:1-OH acylcarnitine	2.71
444.331	2.773	C17:1-DC acylcarnitine	3.38
444.367	2.911	C18:0-OH acylcarnitine	2.25
448.341	2.901	C20:4 acylcarnitine	3.79
452.373	2.977	C20:2 acylcarnitine	2.21
454.316	2.706	C18:3-DC acylcarnitine	1.98
454.388	3.037	C20:1 acylcarnitine	3.52
456.331	2.725	C18:2-DC acylcarnitine	3.29
456.404	3.099	C20:0 acylcarnitine	2.84
458.348	2.726	C18:1-DC acylcarnitine	0.44
458.348	2.76	C18:1-DC acylcarnitine	0.44
512.466	3.52	C24:0 acylcarnitine	3.71

[a] GNPS library match (see **Figure S5a**).

Table S2. Instrumental and LC-MS data processing methods.

**Instrumental methods**			
Use Lock Masses	Off		
Chromatogram Peak Width	6 seconds		
Method Duration	7.50 min (GI tract), 12.50 min (Plasma and cell culture)		
Exclusion list	371.101		
	235.206		
	311.084		
	314.131		
	285.0135		
	144.9822		
**LC parameters**			
Tissue samples			
**Time (min)**	**Flow (mL/min)**	**%B (Acetonitrile + 0.1% Formic Acid)**	**Curve**
0.00	0.500	2	5
1.00	0.500	2	5
2.50	0.500	98	5
4.50	0.500	98	5
5.50	0.500	2	5
7.50	0.500	2	5
7.50	Stop Run		
Plasma samples and cell culture samples			
**Time (min)**	**Flow (mL/min)**	**%B**	**Curve**
0.00	0.500	5	5
1.00	0.500	5	5
9.00	0.500	100	5
11.00	0.500	100	5
11.50	0.500	5	5
12.50	0.500	5	5
12.50	Stop Run		
**Divert valve parameters**			
Switch at	0.2 min		
**MS parameters**			
Source			
Sheath Gas Flow Rate	35 L/min		
Aux Gas Flow Rate	10 L/min		
Sweep Gas Flow Rate	0 L/min		
Spray Voltage	3.80 (+) / 3.0 (-) kV		
Capillary Temperature	320ºC		
S-lens RF Level	50.0		
Aux Gas Heater Temperature	350.0ºC		
Full MS			
Resolution	17,500 (cell culture samples), 70,000 (all other samples)		

AGC Target	1E6 (GI tract, plasma, cell culture), 3E6 (carnitine treatment tissue samples)
Maximum IT	100 milliseconds (cell culture samples), 246 milliseconds (all other samples)
Scan Range	70 to 1050 *m/z* (GI tract, plasma), 100 to 1500 *m/z* (carnitine treatment tissue samples, cell culture samples)
dd-MS^2	
Resolution	17,500
AGC Target	5E5 (cell culture samples), 2E5 (GI tract, plasma), 1E5 (carnitine treatment tissue samples)
Maximum IT	100 milliseconds (cell culture samples), 54 milliseconds (all other samples)
Top N	5
Isolation Window	3 *m/z* (cell culture samples), 1.0 *m/z* (all other samples)
Fixed First Mass	---
(N)CE/ Stepped	NCE: 20, 40, 60
dd Settings	
Min. AGC Target	8.00E3
Intensity Threshold	8.0E4 (cell culture samples), 1.5E5 (all other samples)
Apex Trigger	2 to 15 seconds (cell culture samples), --- (all other samples)
Charge Exclusion	unassigned (cell culture samples), --- (all other samples)
Peptide Match	--- (cell culture samples), preferred (all other samples)
Exclude Isotope	On
Dynamic Exclusion	5 seconds (cell culture samples), 10.0 seconds (all other samples)

**Data analysis method (MZmine2)**			
**GI tract samples and C2C12 treatment samples**			
		**Polarity**	
		Positive	Negative
**MS^1**	Noise Level	2.0E6	2.0E5
**MS^2**	Noise Level	1.0E3	1.0E3
**Chromatogram Builder**	Mass List	masses	masses
	Minimum Time Span (min)	0.01	0.01
	Minimum Height	6.0E6	5.0E5
	m/z Tolerance (ppm)	10.0	10.0
**Chromatogram Deconvolution**	Algorithm	Baseline Cut-off	Baseline Cut-off
	m/z Range for MS^2 Scan Pairing (Da)	0.01	0.01
	RT Range for MS^2 Scan Pairing (min)	0.2	0.2
	Minimum Peak Height	6.0E6	5.0E5
	Peak Duration Range (min)	0.01 – 3.50 (tissue samples), 0.01-1 (C2C12 samples)	0.01 – 3.50
	Baseline Level	2.0E6	2.0E5
**Deisotoping**	*m/z* Tolerance (ppm)	10.0	10.0
	Retention Time Tolerance (min)	0.5 (tissue samples), 0.05 (C2C12 samples)	0.05
	Monotonic Shape	Checked	Checked
	Maximum Charge	3	3
	Representative Isotope	Lowest *m/z*	Lowest *m/z*
**Alignment**	*m/z* Tolerance (ppm)	10.0	10.0
	Weight for *m/z*	1	1
	Weight for Retention Time	1	1
	Retention Time Tolerance (min)	0.5 (tissue samples), 0.2 (C2C12 samples)	0.5
**Row Filtering**	Minimum Peaks in a Row	6 (tissue samples), 3 (C2C12 samples)	6

	Retention Time (min)	0.20 – 6.45 (tissue samples), 0.2-12.26 (C2C12 samples)	0.20 – 6.45
	Keeps Only Peaks with MS^2 Scans	Checked	Checked
	Reset Peak No. ID	Checked	Checked
**Gap Filling (C2C12 samples only)**	Intensity Tolerance	75%	-
	Retention Time Tolerance	0.2	-
	RT Correction	Checked	-
	m/z Tolerance (ppm)	10.0	-
**Mouse carnitine treatment samples**			
		**Polarity**	
		Positive	
**MS^1**	Noise Level	2.0E5	
**MS^2**	Noise Level	1.0E3	
**Chromatogram Builder**	Mass List	masses	
	Minimum Time Span (min)	0.01	
	Minimum Height	3.0E5	
	m/z Tolerance (ppm)	10.0	
**Chromatogram Deconvolution**	Algorithm	Local Minimum Search	
	m/z Range for MS^2 Scan Pairing (Da)	0.01	
	RT Range for MS^2 Scan Pairing (min)	0.2	
	Chromatographic Threshold	5%	
	Search Minimum in RT range	0.1	
	Minimum Relative Height	5%	
	Minimum Absolute Height	4E5	
	Minimum ratio of peak top/edge	1	
	Peak Duration range (min)	0.01 -3.5	
**Deisotoping**	*m/z* Tolerance (ppm)	10.0	
	Retention Time Tolerance (min)	0.5 (plasma), 0.05 (tissue)	
	Monotonic Shape	Checked	
	Maximum Charge	3	
	Representative Isotope	Lowest *m/z*	
**Alignment**	*m/z* Tolerance (ppm)	10.0	
	Weight for *m/z*	1	
	Weight for Retention Time	1	
	Retention Time Tolerance (min)	0.1	
**Row Filtering**	Minimum Peaks in a Row	4	
	Retention Time (min)	0.20 – 12.00 (plasma), 0.2-6.45 (tissues)	
	Keeps Only Peaks with MS^2 Scans	Checked	
	Reset Peak No. ID	Checked	
**Gap Filling**	Intensity Tolerance	5%	
	Retention Time Tolerance	0.1	
	RT Correction	Checked	
	m/z Tolerance (ppm)	10.0	

Data file S1. Impact of infection on GI metabolite profiles, including infection-modulated metabolites identified through random forest analysis.
